# Natural Allelic Variations in Highly Polyploidy *Saccharum* Complex

**DOI:** 10.3389/fpls.2016.00804

**Published:** 2016-06-08

**Authors:** Jian Song, Xiping Yang, Marcio F. R. Resende, Leandro G. Neves, James Todd, Jisen Zhang, Jack C. Comstock, Jianping Wang

**Affiliations:** ^1^Agronomy Department, University of FloridaGainesville, FL, USA; ^2^College of Life Sciences, Dezhou UniversityDezhou, China; ^3^RAPiD Genomics LLCGainesville, FL, USA; ^4^Sugarcane Research Unit, United States Department of Agriculture-Agricultural Research ServiceHouma, LA, USA; ^5^Sugarcane Field Station, United States Department of Agriculture-Agricultural Research Service, Canal PointFL, USA; ^6^Center for Genomics and Biotechnology, Haixia Institute of Science and Technology, Fujian Agriculture and Forestry UniversityFuzhou, China; ^7^Plant Molecular and Biology Program, Genetics Institute, University of FloridaGainesville, FL, USA

**Keywords:** *Saccharum* complex, sugarcane, target enrichment sequencing, polyploid, allelic variations, SNP(s)

## Abstract

Sugarcane (*Saccharum* spp.) is an important sugar and biofuel crop with high polyploid and complex genomes. The *Saccharum* complex, comprised of *Saccharum* genus and a few related genera, are important genetic resources for sugarcane breeding. A large amount of natural variation exists within the *Saccharum* complex. Though understanding their allelic variation has been challenging, it is critical to dissect allelic structure and to identify the alleles controlling important traits in sugarcane. To characterize natural variations in *Saccharum* complex, a target enrichment sequencing approach was used to assay 12 representative germplasm accessions. In total, 55,946 highly efficient probes were designed based on the sorghum genome and sugarcane unigene set targeting a total of 6 Mb of the sugarcane genome. A pipeline specifically tailored for polyploid sequence variants and genotype calling was established. BWA-mem and sorghum genome approved to be an acceptable aligner and reference for sugarcane target enrichment sequence analysis, respectively. Genetic variations including 1,166,066 non-redundant SNPs, 150,421 InDels, 919 gene copy number variations, and 1,257 gene presence/absence variations were detected. SNPs from three different callers (Samtools, Freebayes, and GATK) were compared and the validation rates were nearly 90%. Based on the SNP loci of each accession and their ploidy levels, 999,258 single dosage SNPs were identified and most loci were estimated as largely homozygotes. An average of 34,397 haplotype blocks for each accession was inferred. The highest divergence time among the *Saccharum* spp. was estimated as 1.2 million years ago (MYA). *Saccharum* spp. diverged from *Erianthus* and Sorghum approximately 5 and 6 MYA, respectively. The target enrichment sequencing approach provided an effective way to discover and catalog natural allelic variation in highly polyploid or heterozygous genomes.

## Introduction

Sugarcane (*Saccharum* spp.) is grown in over 100 countries, mainly in tropical and sub-tropical areas of the world. Recently, sugarcane was considered as a sustainable feedstock for next generation cellulosic biofuel production (Tew and Cobill, 2008). The genus *Saccharum* is considered to include six species, namely *S. officinarum*, *S. spontaneum*, *S. sinense*, *S. robustum*, *S. barberi*, and *S. edule* with tremendous diversity in morphology, genome structure, and composition (D’Hont et al., 1998). *Saccharum officinarum* (2n = 8x = 80) is the primary sugar producing species, which has been domesticated from wild species *S. robustum* (2n = 6x–8x = 60–80) (Grivet et al., 2006). *S. spontaneum* is autopolypoid (2n = 4x–16x = 32–128) with excellent stress tolerance (Roach, 1989; D’Hont et al., 1998). *S. barberi* and *S. sinense* have been considered as interspecific hybrids between *S. officinarum* and *S. spontaneum* following by a series of mutations in India and China, respectively (Grivet et al., 2006). Modern sugarcane cultivars are allopolyploids and interspecific hybrids mostly derived from crosses between domesticated *S. officinarum* and wild species *S. spontaneum*, followed by a series of backcrosses with *S. officinarum.* For example, the French sugarcane cultivar, R570, has a chromosome number of 115 (2n = 115) with 80% of the chromosomes from *S. officinarum* species, 10% from *S. spontaneum* and 10% recombination of these two species (D’Hont et al., 1996). The *Saccharum* genus together with a few other related genera, such as *Erianthus*, *Miscanthus* are referred to as *Saccharum* complex. In some sugarcane breeding programs, beside the *S. officinarum* and *S. spontaneum*, other *Saccharum* spp. such as *S. robustum*, and a few *Erianthus* spp. (2n = 2x–6x = 20–60) in the *Saccharum* complex were used as breeding materials to broaden the genetic base and increase the stress tolerance of modern sugarcane cultivars (Tsuruta et al., 2012; Moore et al., 2014).

A large amount of natural phenotypic variations exist in the *Saccharum* complex (Nayak et al., 2014; Todd et al., 2014). Understanding genomic variation within *Saccharum* complex can provide insights into morphology, environmental adaption, polyploidization, sugar and fiber accumulation, stress tolerance, and other important traits. However, genetic and genomic studies of *Saccharum* complex have been daunting due to its high polyploidy, high heterozygosity, large amount of repetitive sequences, aneuploidy, and large genome size varying from 3.36–12.64 GB (Zhang et al., 2012). Despite significant efforts by several international research groups, a reference genome for sugarcane is still unavailable. However, there are abundant expressed sequence tag (EST) databases generated for sugarcane (Vettore et al., 2003; Hotta et al., 2010), as well as cDNA databases (de Setta et al., 2014), which can be used for gene discovery and characterization. The genome of sorghum (Paterson et al., 2009), the closest relative of sugarcane with a genome sequence available, has been extensively used as reference for sugarcane genomic studies due to its extensive collinearity with sugarcane in the genic regions (Ming et al., 2006; Wang et al., 2010). However, applications of the sorghum genome and the ESTs are still limited for studies requiring polyploid allelic dosage, heterozygous genotypes, and particular sequence variation in the *Saccharum* complex. Genome-wide, reliable, and powerful genotyping tools for *Saccharum* complex are critical for a thorough evaluation of the genetic and genomic variation of *Saccharum* complex polyploidy and for identifying specific alleles underpinning phenotypic diversity and the traits of interests across the entire genome and the entire species.

Single nucleotide polymorphisms (SNPs), presence-absence variation (PAV), copy number variation (CNV) and insertions and deletions (InDels) are abundant in plant genomes and many are functionally relevant (Saxena et al., 2014). These variations have been utilized in many model species and crop plants for high throughput genotyping and used in the assessment of genetic diversity, population structure, linkage analysis, quantitative trait loci (QTLs) mapping, association mapping, and genomic selection (Varshney et al., 2009; Saxena et al., 2014). However, genotyping in highly heterozygous polyploid species, such as sugarcane is much more challenging due to the existence of hom(oe)ologs for most of the loci, and even more complicated, by the interference of close paralogs. A typical gene in a sugarcane clone can have up to 12 different alleles per locus. Therefore, to genotype polyploid plants with less ambiguity, we require a system that can distinguish among different allele variations and precisely infer the allele copy number. Large-scale characterization of SNPs and other sequence variation in the genomes of *Saccharum* complex could provide insight into global pattern of intra-genotype and inter-genotype sequence variation and their potential impacts on gene functions. Further development of these sequence variation into powerful tools for genotyping polyploid *Saccharum* complex will help in sugarcane genetic studies, specifically in exploring and utilizing the genetic resources for crop improvement.

Target enrichment methods combined with next generation sequencing (NGS), in which genomic regions of interest are captured for subsequent sequencing, is an effective approach to investigate allelic variation across a large number of samples, specifically for species with complex genomes. The hybridization based enrichment relies on the probe sequence homology or similarity to fish out the target DNA fragments for enrichment. Typically, if the sequence similarity of the target region is more than 80% with the probe oligo sequences, the target regions will be captured, thus all possible allele variants, even paralogous allele variants will be included in the enriched samples, achieving high sensitivity in retrieving target fragments. Therefore, the hybridization based enrichment is suitable for polyploids and heterozygous *Saccharum* species to capture multiple alleles from a large number of diverse samples. It requires a careful downstream sequence analysis pipeline to exclude non-specific target sequences. Target enrichment combined with NGS has been effectively used for detecting SNPs in several other plant species with complex genomes, including maize (Fu et al., 2010), wheat (Winfield et al., 2012; Jordan et al., 2015), and pine (Neves et al., 2013). In sugarcane, target regions of two clones, one *S. officinarum* and the other *S.* hybrid; spanning 5.8 Mb, enriched through solution-based hybridization have been sequenced (Bundock et al., 2012). However, the application of target enrichment approach across the whole scope of *Saccharum* complex accessions, the evaluation of different sequence analysis pipelines tailored to polyploid species with different ploidy level, calling for various variants and possible haplotypes, and divergence among the *Saccharum* complex were not fully addressed. Deep sequencing the target genomic regions of a diversity panel from *Saccharum* complex would generate a catalog of allelic variance and provide a solid foundation for the development of a platform for high throughput genotyping in *Saccharum* complex.

In this study, we applied a target enrichment sequencing approach to sequence genomic regions spanning a total of 6 Mb of 12 accessions, representing different species in *Saccharum* complex. Here, we describe the sequencing results, several types of sequence variants within and among 12 accessions, genotypes of each accession across 1,100,040 total SNP loci, haplotypes of all target regions, and level of divergence among the represented accessions. This study illustrates the value of target enrichment sequencing in calling allelic variations in polyploid species for accurate genotyping, offering an overview of the genomic variation, and molecular evolution among different accessions of *Saccharum* complex. The data generated in this study would be a useful public resource and catalog of diverse sequence variances among *Saccharum* complex.

## Materials and Methods

### Plant Material

A total of 12 diverse accessions representing different species were selected from a diversity panel of 300 accessions (Nayak et al., 2014) grown at the United States Department of Agriculture (USDA), Agricultural Research Service (ARS) Sugarcane Field Station at Canal Point, FL with three replicates in a split plot design. The diversity panel was a representative sub-collection of the World Collection of Sugarcane and Related Grass (WCSRG). The 12 selected accessions include two accessions of *S. officinarum* (P-MAG-84, NG96-024), two accessions of *S. spontaneum* (SES196, IND81-14), one accession of *S. barberi* (Pathri), one accession of *S. robustum* (NG57-054), one accession of *S. sinense* (TekchaOk), one accession of *E. procerum* (Kalimpon), one accession of *E. rufipilus* (US57-060), one accession of unknown species (US61-037), and two accessions of *S*. spp. hybrids (Q050, R570) (Supplementary Table S1, Supplementary Data).

### Probe Design for Target Enrichment Sequencing

For target enrichment of the 12 accessions, we designed a set of 55,946 probes, which are 120-nucleotide long (Supplementary Table S2). Three different genomic resources were used for the probe design in a strategic way. First, a set of 6,035 probes were designed from 5,546 tentative sugarcane unigenes downloaded from the Gene Index repository Version 3^1^. To identify exon–exon boundaries, we searched the corresponding genes in the Sorghum genome v3.1 (downloaded from Phytozome) using BLAST version 2.2.31 (Paterson et al., 2009). The sugarcane unigenes were realigned to the full-length sorghum genes using GMAP v20141216 (Wu and Watanabe, 2005). Based on the output, putative intronic regions in the unigene sequences were masked and avoided in the probe design. All possible probes in non-masked regions were designed. Subsequently, we selected probes with no hit to sugarcane chloroplast or mitochondria reference genome and with a single unique hit to the sorghum reference genome. The second set of probes comprised of 43,910 probes and consisted of well-distributed regions on the sorghum genome, which contained at least one sugarcane short read sequence (NCBI, accession SRX125303 and SRX142056, Bundock et al., 2012) aligned to it. Regions in the sorghum genome that did not have a valid alignment from the sugarcane reads were masked and not considered in the probe design. Regions of the genome that had high depth were also masked. The genome was divided into 15 kb windows, and similar to the first probe set, all possible probes were designed based on the sequences of the sugarcane short reads that aligned to the non-masked regions of the sorghum genome. Probes were only selected for synthesis if they were unique in the sorghum genome and had no match against chloroplast or mitochondria genomes. The third probe set comprised of 6,001 probes targeting 406 candidate genes involved in biomass, sugar metabolism, and disease resistance (Supplementary Table S2). The biomass related genes comprise of cell wall biogenesis gene, architecture genes, and transcription factors among others, that have been functionally characterized in other models species. Sequences of these genes were BLAST against sorghum transcripts from Phytozome database with *E* value >10e^-6^ and alignment length >300 bases to retrieve the corresponding nucleotide sequences. Then the sorghum transcript targets were used for probe design. The probes were synthesized by Agilent^2^.

### Target Enrichment Sequencing

Genomic DNA samples of the 12 accessions were prepared from leaf tissues using the CTAB method according to Wang et al. (2010) with minor modifications. DNA concentration was detected using Quant-iT PicoGreen dsDNA Assay Kit (Life Technologies). For target enrichment DNA sequence approach, 1 μg of genomic DNA of each accession was fragmented to an average size of 400 bp. DNA libraries were constructed by end-repairing the sheared DNA, A-tailing and adapter ligation, bar-coding, and PCR amplification. The 12 barcoded libraries were equi-molarly pooled to a total of 750 ng for downstream target enrichment capturing. Target enrichment of the 12 accessions was performed following SureSelect Target Enrichment System (Agilent Technologies). The pooled samples were hybridized with biotinylated baits twice to target-specific capture library following manufacturer’s guidelines. After the first and the second hybridization process, the selected libraries were re-amplified through 5 and 12 PCR cycles, respectively. The enriched products were sequenced using Illumina HiSeq 2000 to generate single-end 100-bp reads.

### Sequence Trimming and Mapping

The raw reads were de-multiplexed using Illumina pipeline. Adapter contamination and reads of low quality were identified and removed. Each library was sequenced to a depth providing an approximately 100-fold on targeted regions. The 100-base single-end reads were further filtered based on sequence quality and the barcodes from original reads were trimmed off using Trimommatic v0.36 (Bolger et al., 2014). The reads with quality score ≥20, length ≥50, and free of barcode or primer sequences were kept for further analysis. For the reads alignment, we applied three different aligners, BWA version 0.6.1-r104^3^, Bowtie2 version 2.2.5^4^ and MOSAIK version 2.1^5^ (Li and Durbin, 2010; Langmead and Salzberg, 2012; Lee et al., 2014). The reference genome used here for *S. officinarum* was assembled using SPAdes v3.6.2 (Bankevich et al., 2012) with default settings on reads generated for accession (NG96-024). For the sorghum reference, sorghum genome v3.1 was used. Both of these references were used for alignment using three different aligners with close and comparable settings. Bowtie 2 (v2.2.5) was used with the option -sensitive-local. MOSAIK was applied with the following parameters: mismatches (-mmp 5), hash size (-hs 15), -mfl 500. BWA-mem was run following default settings. Uniquely mapped reads for each accession were extracted by using “XS:I” and “ZA” tag indicating secondary alignment in BAM file for Bowtie2 and MOSAIK, respectively. For BWA-mem, uniquely mapped reads with mapping quality >0 were extracted from sequence alignment BAM files.

To evaluate the best reference of the reads from *Saccharum* species beside the sorghum genome v3.1, the assembly of all sequence reads (>200 bp), probe sequences, and a unigene set assembled from in-house RNA-seq data were also used as the reference for alignment and comparison. For the RNAseq unigene set assembling, Trinity v2.1.1^6^ with default settings was used. Assembled contigs with more than 200 bases were used as the reference for alignment (Haas et al., 2013).

### Characterization of Probe Features and Capture Efficiency

For probe feature characterization, the probes with at least five sequence reads capture were selected for investigation of thermodynamic parameters. The GC content for each probe was calculated using custom Perl scripts. Probe hybridization free energy (PHFE), hairpin, and dimer scores were calculated using Python scripts according to Xia et al. (2010). The hairpin and dimer scores were calculated with default setting, and the PHFE script was run with RNA as nucleic acid and temperature at 65°C. The melting temperature (Tm) was calculated by using MELTING software with the following parameters: -B RNA/DNA hybridization, -A Sugimoto et al., 1996, -N 1 and -P 0.0001 (Le Novere, 2001). The probe sequence minimum folding energy (PMFE) was analyzed by metl.pl script (Markham and Zuker, 2008).

To assess the probe capture efficiency and the captured sequence depth on the target region, we aligned all the probe sequences against the sorghum genome with BLAT v20140318 (alignment identity >102) (Kent, 2002). A bed file was generated containing target capture region by adding 100-bp up and downstream of each probe on the sorghum reference sequences. Bedtools coverage (v2.23.0) was used to calculate the sequencing depth in a bam file containing read alignments and a bed file containing target capture regions (Quinlan and Hall, 2010). The cumulative distribution describing the fraction of target region was plotted in R program using ggplot2 package (Wickham, 2009). To assess the efficiency of each probe, we aligned the reads generated from each accession against each probe and created sorted BAM files. The cumulative sequencing depth on each probe was plotted using the same approach.

To understand the relationship between probe features and probe capture efficiency, we established a linear model between uniquely mapped reads captured by the probes and the probe thermodynamic parameters by using *S. officinarum* (NG96-024). Only the probes with >26 (10% of the average uniquely mapped reads/probe) and <974 uniquely mapped reads (99th percentile of the distribution of the uniquely mapped reads/probe) were considered as successful probes to reduce potential bias due to sequence difference between designed probe sequences and target regions. The correlation coefficients between thermodynamic parameters were estimated using R 3.0.2 (R Development Core Team, 2010). If parameters have correlation coefficient higher than 0.95 or lower than -0.95, then only one parameter from this correlation group was kept for a model construction. A linear model was constructed between the capture efficiency and representative thermodynamic parameters using a linear regression analysis on R 3.0.2 (R Development Core Team, 2010). Only the parameters significantly contributing [tested using Analysis of variance (ANOVA)] to the capture efficiency were kept in the model. Further, the importance of the parameters kept in the model was evaluated using Relaimpo package in R 3.0 (Groemping, 2006).

### Sequence Variants and Haplotype Calling

Different reference sources and aligners were compared to establish the optimized pipeline for sequence analysis within *Saccharum* complex. For SNP calling, each accession alignment SAM file was converted into BAM file followed by binary compressing, sorting, and indexing using SAMtools (samtools view, sort, and index tools). SNPs and InDels were called with three different variant callers, namely, Samtools (v 0.1), UnifiedGenotyper implemented in Genome Analysis ToolKit (GATK) v3.30, and Freebayes v0.9.21 respectively (Li et al., 2009; Garrison and Marth, 2012; Auwera et al., 2013). Samtools was used to call variants with the parameters -u -D -g -f, Freebayes was run with the parameters -C 2 -F 0.05 -0 -q 20 –min-coverage 5 -p (ploidy level) and GATK was used with settings: -ploidy (ploidy level) -mbq 20 -mgl 20-stand_call_conf 30 -stand_emit_conf 10. To obtain high-confidence SNP, the GATK specific filters were applied to vcf from GATK. Other than that, a series of stringent filters were applied to the vcf files generated from different callers including; (1) mapping quality for SNPs >30, (2) base quality for SNP sites >20, (3) number of reads for alternate base for each accession > 2, (4) overall maximum depth for 12 accessions <3000, (5) overall minimum depth for 12 accessions quality >600, and (6) minimum value of the QUAL field >80. The filtered SNP variants were annotated with SnpEff (v3.6) program using the sorghum v3.1 reference genome to determine the effects of the SNPs on function of genes. To avoid assigning the SNP to more than one gene model, default settings were used except that the upstream/downstream distance was set at 0 (Cingolani et al., 2012). The synonymous and non-synonymous substitutions were also identified using SnpEff. To increase the accuracy of InDel calling, only the concordant sites among three callers were considered as a potential InDel for the analysis (Albers et al., 2011). Variants (SNPs and InDel) between sorghum reference and any of the 12 accessions were filtered out and only variants within each and between every two of the 12 accessions were used for further analysis. The UnifiedGenotyper was used to assign genotypes at each SNP sites based on the ploidy level. The heterozygotes at each locus including different dosage level such as single dose, double dose, triple dose etc., up to the ploidy level according to their genotypes were called by Unified Genotyper. The heterozygosity rate for each accession was estimated based on the heterozygous loci by dividing number of heterozygous sites/length of aligned region. HapCompass v0.7.7^7^ was used for haplotype assembly with ploidy level assigned by -p (Aguiar and Istrail, 2012). The custom scripts were deposited in Github^8^.

### Validation of SNPs

The SNPs identified above were selected for validation by Sanger sequencing. Primers (Supplementary Table S3) were designed from the flanking sequencing of putative SNPs based on the *de novo* assembly to obtain amplicon size of 500–1000 bases using Primer 3^9^. Five genotypes (TekchaOk, US61-037, US57-060, P-MAG-84 and IND81-14) representing four different species and unknown species were used as template for amplification of the target SNPs. The amplified products were purified and sequenced by ABI 3730 sequencer and the sequencing results were analyzed using DNAbaser 4.20^10^.

### Structural Variation Analysis and Annotations

Structural variations including gene CNV and gene PAV were analyzed following the approach described by Swanson-Wagner et al. (2010). To evaluate the gene CNVs between every two accessions within *Saccharum* species and CNVs between the two *Erianthus* species (Kalimpon and US57-060), the number of uniquely mapped reads to sorghum gene model was recorded for each pair of accessions. The depth of coverage was normalized using the following formula: read depth of gene model = [10^9^× (no. of reads in gene)]/[(Total mapped reads) × (length of gene)] to eliminate the effect of read depth of each sample. For each gene, the log_2_ ratio of read depth of query accession to subject accession was used to assess CNV. To reduce technical variation between two accessions, we used 99th percentile cut-off. Up-CNVs were identified as above 99th percentile cut-off in query accession, and Down-CNVs were identified as below 99th percentile cut-off in subject accession. The gene PAVs were identified between every two accessions which met the criteria including; (1) no reads aligned to query accession; and, (2) at least 10 reads aligned to subject accession at the same gene region with normalized read depth of no less than 0.1.

To annotate the genes with CNV or PAV, the GO terms for each gene were retrieved from sorghum genome^11^. The GOslim annotation of genes that were involved in the structural variation including CNVs and PAVs was performed using agriGO^12^ (Du et al., 2010). The GOslim terms were calculated by agriGO by using Fishers testing method with a threshold FDR corrected *P*-value <0.05.

### Sequence Divergence Analysis

The clean reads of each accession were *de novo* assembled using SPAdes v3.6.0 (Bankevich et al., 2012) with default settings to identify the coding DNA sequences (CDS). The assembly of each accession representing the consensus sequence was used to estimate the divergence among the 12 accessions and sorghum. Sorghum CDS were downloaded from Phytozome^13^ and pairwise alignments of the CDS were conducted using CLUSTALW v2.1^14^ (Li, 2003). The substitution rates of synonymous (Ks), non-synonymous (Ka), and Ka/Ks ratio were calculated using the maximum likelihood algorithm developed by Nei and Gojobori (1986) and implemented in PAML v4.9a (Yang, 2007). The common synonymous substitution rate of 6.9 × 10^-9^ for grass lineage was used to estimate the divergence time (Gaut et al., 1996). The phylogenetic tree was constructed by using bi-allelic SNP among 12 accessions without missing data. Phylip v3.696 was used for phylogenetic analysis implemented with seqboot (1000 replicates) bootstrapping. The treefile was generated using Dnapars (Felsenstein, 2002) and the generated treefile was visualized with FIGTREE v1.4.0^15^.

## Results

### Sequences Generated from Target Enrichment

**FIGURE 1:**
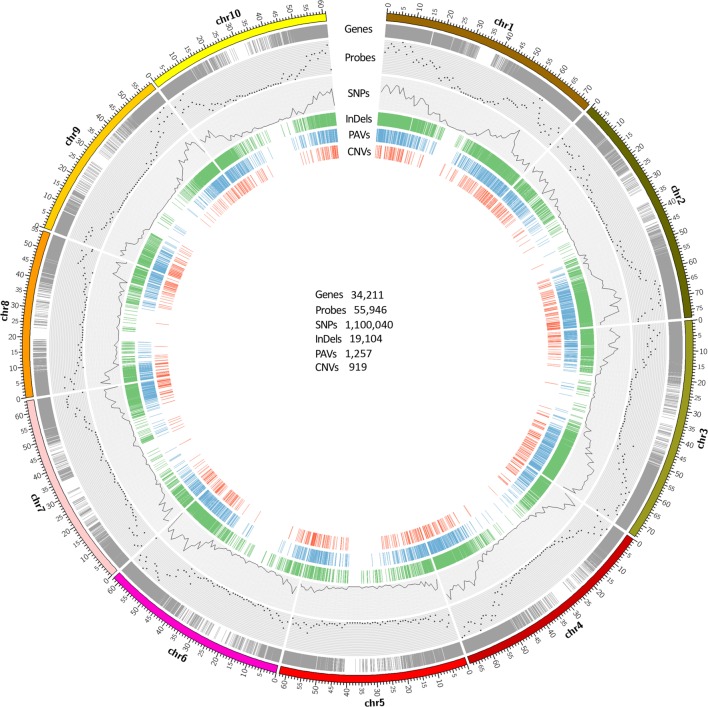
**Genome-wide distribution of probes and sequence variants along sorghum genome.** Gene models are displayed by gray ring showing the majority of gene regions on the chromosome arms. Scatter plot represents the number of probes within 1 Mb window size across sorghum genome. The SNPs for 12 accessions are plotted by the black line which represents the SNP density within 1 Mb window size across sorghum genome. The green color represents concordant InDels which were called by Samtools, GATK, and Freebayes. The blue and red color represent copy number variations, and presence and absence of variations identified in this study respectively. The number of gene models and each variant category are shownaren in the circle.

A total of 55,946 120-mer probes were designed from sugarcane and sorghum sequences to capture the corresponding target regions of the 12 accessions in *Saccharum* complex. These probes targeted approximately 41.4% of 34,211 sorghum gene models, 3,413 non-genic regions in total spanning an accumulative 6 Mb genomic region. Due to repetitive sequences or duplicative genes, which were not suitable for probe design, some genes were not targeted. These probes were distributed across each arm of sorghum chromosomes (**Figure 1**) with an average of one probe every 13 kb of the sorghum genome. Sequencing of fragments captured by the probes from the 12 accessions generated a total of 488.1 million 100-base single end reads ranging from 22.8 to 56.7 million reads with an average of 40.7 million reads per accession. The raw data was deposited in NCBI SRA with accession number PRJNA300644. There were 411.7 (84.3%) million clean reads left after quality control and adaptor trimming. These clean reads comprise of 97 Gb of high quality sequences with an average read length of 87 bases (**Table 1**, GenBank: SRP065668). The uniquely mapped reads covered approximately 27 Mb of intron region and 26 Mb of exon region of the sorghum reference genome.

**Table 1:** Statistics of target sequencing reads alignment to four different sequence sources as reference.

			Sorghum genome		Probes		EST unigene set		Assembly (>200 bp)	
	No. of raw reads (millions)	No. of clean reads (millions)	No. of reads mapped (millions)	Percentage of reads mapped (%)	No. of reads mapped (millions)	Percentage of reads mapped (%)	No. of reads mapped (millions)	Percentage of reads mapped (%)	No. of reads mapped (millions)	Percentage of reads mapped (%)
NG96-024 (S. o)	28.4	25.6	21.9	85.6	13.8	53.9	18.1	70.7	18.0	70.5
P-MAG-84 (S. o)	46.5	40.2	34.3	85.5	21.2	52.9	28.3	70.5	28.2	70.4
IND81-14 (S. sp)	52.2	42.0	35.2	83.9	18.2	43.4	28.9	68.9	29.1	69.4
SES196 (S. sp)	35.4	28.4	24.0	84.3	12.6	44.2	19.9	69.8	19.8	69.6
Pathri (S. b)	40.7	36.5	31.5	86.3	20.3	55.6	25.8	70.6	25.7	70.4
NG57-054 (S. r)	35.5	31.4	27.4	87.1	18.7	59.5	22.5	71.5	22.2	70.6
TekchaOk (S. si)	44.9	35.9	30.3	84.4	17.5	48.8	25.0	69.7	25.1	69.8
Q050 (S. h)	56.7	47.5	40.4	85.1	22.5	47.5	33.3	70.3	33.3	70.2
R570 (S. h)	45.8	35.5	30.5	86.0	16.1	45.5	24.8	69.7	25.1	70.7
US61-037 (un)	55.2	48.5	42.7	88.0	26.7	55.1	34.2	70.6	34.3	70.7
Kalimpon (E. p)	22.8	19.7	16.1	81.9	9.4	47.7	12.4	62.8	13.6	68.9
US57-060 (E. r)	24.0	20.5	17.1	83.5	9.8	47.9	13.1	64.0	14.1	68.7
Total	488.1	411.5	351.3		206.9		286.2		288.5	
Average	40.7	34.3	29.3	85.1	17.2	50.2	23.8	69.1	24.0	70.0

*S. o, *Saccharum officinarum*; S. sp, *Saccharum spontaneum*; S. b, *Saccharum barberi* S. r, *Saccharum robustum*; S. si, *Saccharum sinense*; S. h, *Saccharum* hybrid; un, unknown; E. p, *Erianthus procerum*; E. r, *Erianthus* rufipilus.*

The captured fragments also contain targeted genomic sequences homologous to the probes along with their flanking regions. Hence, the overall sequenced reads covered longer regions than the size of the intended targets (Supplementary Figure S1). To assess capture efficiency and determine if we obtained sufficient reads for rare allele detection, sequencing depth across all target regions including probe and their flanking regions were evaluated for all 12 accessions based on the sorghum genome. The median sequence depth (MSD) varied between different species. The capture efficiency in *Saccharum* species was slightly higher than that in *Erianthus* species, since *Saccharum* species is genetically closer to sorghum than *Erianthus* species and sorghum genome was used as a reference for the alignment of the sequence reads. For all *Saccharum* species, 60% target regions had about 50 × MSD, while less than 50% target region had 50 × MSD in *Erianthus* species (Supplementary Figure S2).

### Characterization of Probes for Target Enrichment

To investigate the capture efficiency of individual probes, the sequence of each probe was used as reference for reads mapping. For each individual accession, approximately 90% of the probes captured reads with read depth >30x and 50% of the probes with read depth >100x (Supplementary Figure S2), indicating an overall success of probe design and target enrichment. However, the efficiency of probes is highly uneven with 593 probes showing MSD above 10,000, 1,783 probes with MSD below 100, and 421 probes failing to capture any fragments.

To determine the features and probes with good capture efficiency, several probe sequence parameters were investigated including Tm, probe sequence minimum folding energy (PMFE), hybridization folding energy (PHFE), hairpin score, and dimer score (Supplementary Figures S3A–F). The capture efficiency reflected by MSD was higher for probes designed from CDS region of sorghum genes than those from non-CDS region (Supplementary Figure S3G). The capture efficiency was slightly higher for probes derived from sugarcane sequences compared to those from sorghum sequences (Supplementary Figure S3H). The optimal MSD was observed for probes with GC content ranging from 30–60%. The hairpin score shows a similar effect on MSD compared to that of dimer score (Supplementary Figure S3A).

The successful probes (50,820) for *S. officianrum* (NG96-024) were used to construct a model for explaining the probe efficiency. For the simplicity of this model, we tentatively assume that the probe sequences have equal copy numbers in the genomes. The GC content of probe sequence was highly correlated with Tm and PHFE. Therefore, only GC content was chosen from this correlation group in combination with other three parameters, hairpin, dimer scores and PMFE used for the model construction. The ANOVA results indicated that these four thermodynamic parameters significantly (*p* < 0.001) contributed to uniquely mapped reads of probes, from which a linear regression model was constructed as: Uniquely mapped reads = 5.08^∗^GC content + 13.3^∗^PMFE -2.11^∗^Dimer – 10.67^∗^Hairpin + 164.46. Based on the established model, higher GC content and PMFE could lead to more uniquely mapped reads, while Dimer and Hairpin had negative effects on the uniquely mapped reads or capture efficiency. The importance of the four parameters, PMFE, GC, Hairpin, and Dimer, showed a decreasing order (Supplementary Figure S3I).

### Alignment of Target Enrichment Sequences

To select the best aligner for efficiently calling sequence variants, three most popular aligners for NGS read alignment, Bowtie2, BWA-mem, and MOSAIK were compared by aligning the cleaned reads from a single library to its assembly and sorghum genome respectively. The portion of mapped reads using Bowtie2, BWA-mem, and MOSAIK with less than 5 mismatches were 92.8, 92.3, and 87.4%, respectively. Both Bowtie2 and MOSAIK have less uniquely mapped reads (64.1 and 77%) to contig assembly from NG96-024 than BWA-mem (88.6%) indicating that BWA-mem was more sensitive for the uniquely mapped reads alignment. A follow-up visual examination on randomly selected alignment regions further confirmed that BWA-mem could accurately and sensitively call the uniquely aligned reads. Therefore, BWA-mem was used for all the alignments in this study. To determine the best reference sequence for the alignment (since the sugarcane genome is not available currently) the clean reads of the 12 accessions were aligned to sorghum genome v3.1, probe sequences, assembled sugarcane transcript sequences, and assembly of the total reads (contig length >200 bp) respectively using BWA-mem. The alignment rate using sorghum genome as the reference was 83%, ranging from 80–86% for each accession, the highest in the four reference sources (**Table 1**). Therefore, the sorghum genome was used as a reference for alignment of the reads from different species in *Saccharum* complex.

### Calling Sequence Variants

**FIGURE 2:**
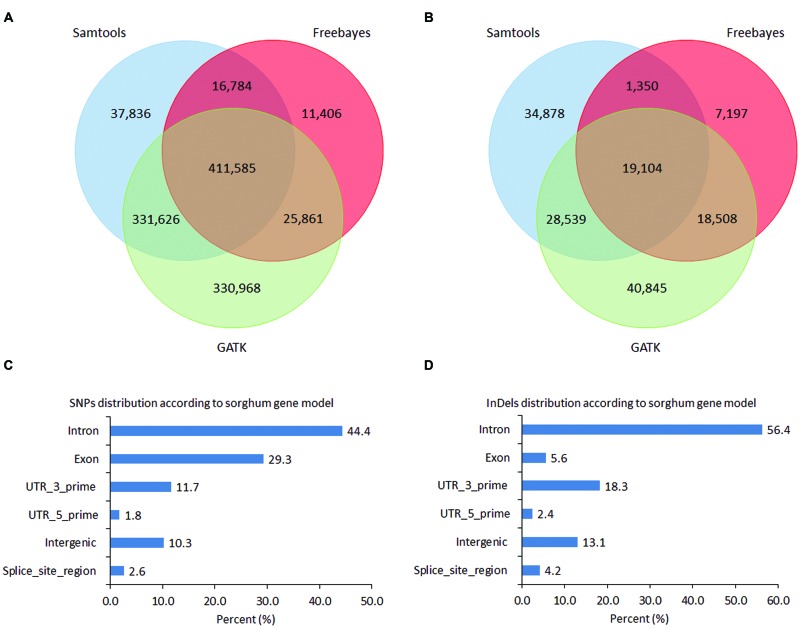
**Number of single nucleotide polymorphisms (SNPs) and InDels.** **(A)** Venn diagram showing overlapping SNP variants among Samtools, GATK, and Freebayes callers; **(B)** Venn diagram showing overlapping InDel variant sites among Samtools, GATK, and Freebayes callers; **(C)** SNPs distribution according to sorghum gene model by using SNP calls from GATK; **(D)** InDels distribution according to sorghum gene model by using common InDels generated by three callers.

Single nucleotide polymorphisms and InDels were called based on unique alignments of cleaned reads to sorghum genome using BWA-mem. To create a set of high-confidence SNPs and InDels, three different callers, namely, SAMtools, GATK, and Freebayes were used. The chromosome number and ploidy level of each accession were estimated (Supplementary Data, Supplementary Table S1) to facilitate the variants calling specifically by using GATK and Freebayes, which can accommodate species with different ploidy level. In total, 797,831, 1,100,040, and 465,636 SNPs were called by using SAMtools, GATK, and Freebayes respectively (**Figure 2A**) with a total of 1,166,066 non-redundant SNPs and 411,585 commonly called SNPs by three callers. In terms of the types of SNP changes, transition (Ts) and transversion (Tv), the Ts/Tv mutation ratios of SNPs produced from SAMtools, GATK, and Freebayes were 1.64, 1.61 and 1.76, respectively. In order to validate the accuracy of SNP calling of these three callers, 13 SNP-containing regions were selected for validation by Sanger sequencing including 69, 31, and 57 SNPs called by Samtools, Freebayes, and GATK respectively. Among those SNPs, 22 were called by all three callers, 47 SNPs specifically called by Samtools, 35 by GATK, and 9 by Freebayes (Supplementary Table S4). Among the 13 primer pairs designed for the SNP-containing regions, 12 could amplify target sequences. Within these amplified sequences, 113 SNPs were validated which gave validation rates of 87% for Samtools-specifically called SNPs, 89.5% for GATK, 90.3% for Freebayes, and 86.4% for the concordantly called SNPs by all the three callers respectively (Supplementary Table S4). The SNPs not validated were mostly single dose markers. In total, 83,871, 106,996, and 46,159 InDels with size ranging from 1 to 20 bp were called by using SAMtools, GATK, and Freebayes respectively with a total of 150,421 non-redundant InDels and 19,104 concordantly called InDels by the three callers (**Figure 2B**).

**FIGURE 3:**
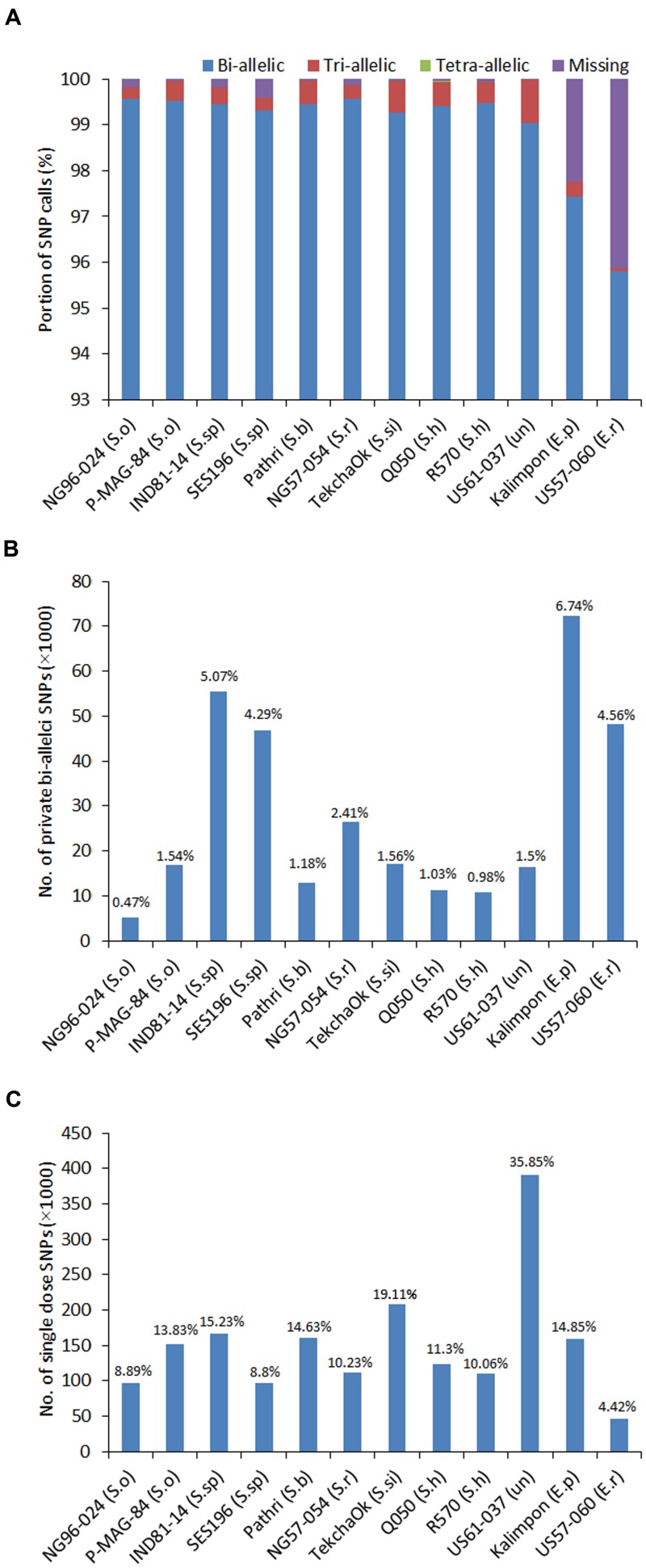
**Distribution of SNP types and genotypes.** **(A)** The percentage of various SNP calls including bi-allelic, tri-allelic, tetra-allelic SNPs and SNP loci with missing data for 12 accessions; **(B)** The percentage of the genotype-specific SNPs called for each accession; **(C)** The number of single-dose markers called for each accession.

To further evaluate the SNP number variation and identify the common SNPs among the 12 accessions, the SNPs called by GATK (1,100,040 in total) were sorted out for each accession. GATK with consideration of ploidy level called more SNPs than FreeBayes and had better accuracy in SNP calling than SAMtools. The average read depth for SNP called by GATK was 1,653. With the read depth increasing, the number of SNPs called decreased and reached to a flat line when the read depth was between 1,500 and 2,900, which might be a reliable depth range for SNP calling. Each of the 12 accessions possessed more than 95% of the SNPs called by GATK. In total, 1,091,239 (99.2%) SNPs were present in all 10 *Saccharum* accessions, suggesting that most SNPs were cross-species differences. The two *Erianthus* accessions, US57-054 and Kalimpon had 95.9 and 97.8% of the total called SNPs with 45,272 (4.1%) and 24,718 (2.2%) of missing SNPs due to lack of reads alignment (**Figure 3A**). Nevertheless, the ploidy level of these 12 accessions, varied from 6 to 12, and the frequency of multi-allelic SNP loci for each single accession was very low (less than 1%) (**Figure 3A**). The number of accession-specific bi-allelic SNPs varied from 5,154 (0.47%) for *S. officinarum* accession (NG96-024) to 72,266 (6.74%) for *E. procerum* accession (Kalimpon) (**Figure 3B**). The SNPs were further estimated according to the types of nucleotide change, transitions or transversions and the Tv/Ts ratio was observed to be lower in the two *Erianthus* species than that in the other *Saccharum* species.

Read depth was used to evaluate gene CNV among the 10 *Saccharum* species and between the two *Erianthus* species. Since several factors can affect the read depth including ploidy level, capture efficiency, read number of each sample, and alignment rate; the read depth was normalized against the ploidy level and number of reads per sample. Accessions within the same genus were grouped together for gene CNV calling to minimize various homology levels between different species which can cause alignment bias. In total, 919 gene CNVs were identified including 118 gene CNVs between two *Erianthus* species and 821 gene CNVs among 10 *Saccharum* species. The largest number of gene CNVs (172) were identified between *S. spontaneum* accession (IND81-14) and *S.* hybrid accession (R570) including 117 up-CNVs and 55 down-CNVs in *S. spontaneum* accession (IND81-14), followed by 137 gene CNVs between *S. officinarum* accession (NG96-024) and *S. spontaneum* accession (IND81-14) including 56 up-CNVs and 81 down-CNVs in *S. officianrum* accession (NG96-024). The smallest number of CNVs (22) was identified between *S. officinarum* accession (P-MAG-84) and *S.* hybrid accession (Q050) including 16 up-CNVs and 6 down-CNVs in *S.* hybrid accession (Q050). Compared to *E. procerum* accession (Kalimpon), 41 up-CNVs and 77 down-CNVs were identified in *E. rufipilu* accession (US57-060) (**Table 2**). PAVs represent the extreme form of down-CNV where copy number became zero in case of absence. A total of 1,257 gene PAVs were identified including 51 gene PAVs between two *Erianthus* species and 1,215 gene PAVs among 10 *Saccharum* species. The largest number of PAVs (222) was identified between *S.* hybrid accession (R570) and *S. spontaneum* accession (IND81-14) including 207 gene deletions in *S.* hybrid accession (R570) and 15 gene deletions in *S. spontaneum* accession (IND81-14), followed by 137 PAVs between *S.* hybrid accession (R570) and *S. barberi* accession (Pathri) including 130 deletions in *S.* hybrid accession (R570) and 7 gene deletions in *S. barberi* accession (Pathri). The smallest number of 14 PAVs was observed between unknown accession (US61-037) and *S. officinarum* accession (P-MAG-84) including 6 presence and 8 absence in unknown accession US61-037 (**Table 2**).

**Table 2:** Summary of copy number variation (CNV) and presence/absence variation (PAV) identified within *Saccharum* spp. and between two *Erianthus* spp.

	NG96-024 (S. o)	P-MAG-84 (S. o)	IND81-14 (S. sp)	SES196 (S. sp)	Pathri (S. b)	NG57-054 (S. r)	TekchaOk (S. si)	Q050 (S. h)	R570 (S. h)	Kalimpon (E. p)
**Query**										
P-MAG-84 (S. o)	34/8 (37/4)									
IND81-14 (S. sp)	81/56 (86/27)	40/60 (23/32)								
SES196 (S. sp)	63/70 (18/65)	29/75 (6/29)	21/67 (7/100)							
Pathri (S. b)	60/8 (52/8)	21/19 (19/22)	53/50 (40/41)	72/20 (111/14)						
NG57-054 (S. r)	24/17 (18/9)	8/27 (3/23)	59/71 (23/59)	66/58 (61/18)	11/57 (5/33)					
TekchaOk (S. si)	45/7 (23/5)	18/15 (6/16)	61/38 (27/48)	70/19 (79/10)	15/12 (13/24)	26/4 (29/7)				
Q050 (S. h)	52/3 (41/1)	16/6 (16/4)	67/34 (52/33)	82/20 (128/5)	25/11 (35/19)	36/11 (36/1)	19/10 (23/6)			
R570 (S. h)	38/8 (11/26)	15/36 (3/102)	55/117 (15/207)	71/55 (49/40)	20/44 (7/130)	13/12 (13/44)	12/23 (3/83)	11/40 (1/129)		
US61-037 (un)	42/5 (32/2)	16/23 (6/8)	67/64 (32/48)	81/48 (98/6)	23/31 (21/26)	31/9 (23/4)	9/22 (12/10)	17/28 (9/18)	22/15 (72/2)	
US57-060 (E. r)	–	–	–	–	–	–	–	–	–	41/77 (12/39)

*Number shows the up-CNVs/down-CNVs (presence/absence) in Query gentoype. S. o, *Saccharum officinarum*; S. sp, *Saccharum spontaneum*; S. b, *Saccharum barberi*; S. r, *Saccharum robustum*; S. si, *Saccharum sinense*; S. h, *Saccharum* hybrid; un, unknown; E. p, *Erianthus procerum*; E. r, *Erianthus* rufipilus*.

### Variants Annotation and Their Effects

All the SNPs called were dispersed across the entire sorghum genome, consistent with the probes distribution showing high density on the euchromatic arm regions and reduced density near the centromeres (**Figure 1**). The largest number of SNPs was distributed on sorghums chromosome 1 (173,809, 15.8%), followed by chromosome 3 (170,064, 15.5%). The smallest number of SNPs was found on chromosome 5 (51,767, 4.7%) followed by chromosome 8 (60,917, 5.5%). To demonstrate the distribution of SNPs relative to the coding regions and their potential impacts on the sequence function, the locations of SNPs called by GATK were projected onto sorghum gene models. In total, 987,237 out of 1,100,040 SNPs called from GATK, might have effects on gene functions due to various locations on the gene models. Out of 811,201 SNPs, 322,533 (29.3%) were located in the exon region and 488,668 (44.4%) were located in the intron region of the gene models. SNP density was lower in exon regions (1 SNP/82 bp) as compared to that in the intron regions (1 SNP/55 bp). The SNPs located on the exon regions included 170,544 non-synonymous and 156,816 synonymous SNPs. SNP sites in the exon regions included 3,519 SNPs introducing premature stop codons, 92 of the SNP sites causing start codon mutation, and 1,558 splice sites. Among the remaining SNPs, 112,803 (10.3%) were located on intergenic regions, and 147,958 (13.4%) were located in the UTRs (**Figure 2C**). In total, 14,035 sorghum gene models contained SNPs showing an average of 71.6 SNPs per gene model ranging from 1 to 1,157 SNPs (in Sobic.003G180100, a callose synthase gene with length of 17,263 bp) per gene model, which resulted in an average SNP density of 1 SNP/15 bp in the genic region (Supplementary Table S5).

In total, 19,104 InDels were consistently called by the three callers and these InDels were distributed widely across the 10 chromosomes of the sorghum genome with an average density of 36 kb/InDel. Based on their location, majority of the InDels (16,601, 87%) might have effects on gene functions. Approximately 6% (1,079) of those InDels were located in exon regions, 56.4% (10,767) in intron regions, 20.7% (3,950) in UTR region, and the rest in exon–intron junctions (**Figure 2D**). In consistence with SNP distribution, InDels density on chromosome 1 (24 kb/InDel) and 3 (25 kb/InDel) was four times higher than that on the chromosome 5 (97 kb/InDel) indicating the genome of certain chromosomes were more active in evolving than others. Furthermore, the abundance of different InDel length was investigated. As InDel size increased, the total number of InDels reduced (Supplementary Figure S4). The InDels causing frame-shift mutations were approximately 48% (522), compared to a slightly lower number for InDels in-frame (557, 52%) (with a length of a multiple of 3 bp in the coding region), indicating a significant evolvement of frame shift InDels with perhaps deleterious effects on functional genes (Supplementary Figure S4A). However, across all regions assayed, the InDels with a length of not a multiple of 3 were much (∼80.9%) more abundant than the InDels with a length of a multiple of 3 (Supplementary Figure S4B), confirming that the InDels in the CDS are indeed subject to purifying selection.

The functional effects of structural variations including CNVs and PAVs were also annotated according to sorghum gene models. Out of 34,213 gene models, 919 had CNVs among the 10 *Saccharum* species and between two *Erianthus* spp, including 98 CNVs accruing only between two *Erianthus* spp. The annotation showed that 397 genes had Gene Ontology (GO) annotation in sorghum genome and 521 genes did not have any GO annotation. Gene family enrichment analysis indicated that genes with CNVs were significantly (*P* < 0.001) enriched in 14 GO terms related to photosynthesis and nucleotide binding (Supplementary Figure S5A). Interestingly, seven down-CNVs showed *S. spontaneum* species-specificity, which indicated that these seven genes, such as NRAMP metal ion transporter family protein (Sobic.001G125400), terpene synthase (Sobic.007G133900), and PR5-like receptor kinase (Sobic.003G097200), had a significantly low copy number in the two *S. spontaneum* accessions but much high copy number in other *Saccharum* spp. (Supplementary Figure S6). Out of the total 1,257 genes showing PAVs between different accessions, 541genes were annotated in sorghum and 716 genes were of unknown function. The GO enrichment analysis showed that 13 GO terms related to oxidative stress and ion binding were significantly represented (Supplementary Figure S5B). In addition, one disease resistance gene, Sobic.006G014632, was absent in *E. rufipilus* accession (US57-060), *E. procerum* accession (Kalimpon), unknown accession (US61-037), *S.* hybrid accessions (R570), *S. robustum* accession (NG57-054), *S. officinarum* accession (P-MAG-84) and *S. officinarum* accession (NG96-024), but present in *S. sinense* accession (TekchaOk), *S. spontaneum* accessions (SES196), *S. spontaneum* accessions (IND81-14) and *S.* hybrid accessions (Q050), which may be associated with different capabilities to handle biotic stress in wild and cultivated sugarcanes.

### Genotype and Haplotype Calling

Genotype calling based on SNPs in polyploids is critical in determining the allele dosage and also is very challenging due to high ploidy level, limited number of nucleotides (four nucleotides only, A, C, T, G), and sometimes insufficient sequence depth or low quality. Since the ploidy levels included 6, 8, and 12 in the 12 accessions, the genotypes were called at different ploidy levels using bi-allelic SNPs for each of the 12 accessions (Supplementary Figures S7A–C). The largest number of genotypes was found as homozygote with reference allele, followed by homozygote with alternative allele regardless of which ploidy level was applied (Supplementary Figures S7A–C). The overall heterozygosity rates of 12 accessions ranged from 0.28% in *E. rufipilus* accession (US57-060) to 0.96% in unknown spp. (US61-037). Among *Saccharum* spp., *S. barberi* (0.52%), *S. sinense* (0.6%) and hybrid R570 have relatively higher heterozygosity rate in target region than the others (Supplementary Table S6). The heterozygotes at each locus including single dose, double dose, triple dose etc. up to the ploidy level was less than 25% in total (except US61-037). The single dose markers (SDM) were calculated based on the ratio of reads calling of each allele and ploidy level for each of the 12 accessions. A total of 999,258 SDM were identified ranging from 46,587 (4.42% of bi-allelic alleles in *E. rufipilus* accession, US57-060) to 390,676 (35.85% of bi-allelic alleles in unknown accession, US61-037) (**Figure 3C**).

To further extract haplotypes of each accession at every single locus, the overlapping reads containing SNPs called at an average depth of 50 was used to reconstruct the haplotypes. The longest haplotype blocks identified in the 12 accessions range from 1,358 bases to 2,453 bases with an average of 1,683 bases showing no big variance among accessions (Supplementary Table S6). The longest haplotype block of 2,453 bases was identified in hybrid accession (R570), which included 183 SNP sites on Chr10 (60,795,871-60,798,323) followed by haplotype block with length of 2,211 bases on Chr05 (64,826,268-64,828,478) in a hybrid accession (Q050). Both blocks were located in the genic region encoding glycosyltransferase gene.

By looking into the haplotype blocks within each of the 406 candidate gene regions, a total of 13,584 haplotype blocks were inferred ranging from 394 in *S. officinarum* accession (P-MAG-84) to 1,493 in unknown accession (US57-060). In general, the number of haplotype blocks and the SNP density of single candidate gene increased with increasing number of uniquely mapped reads (Supplementary Figures S7D,E). However, all the possible haplotypes could be resolved when the uniquely mapped read number reaching a certain level (Supplementary Figure S7F). The haplotype block number also increased with the SNP density of each candidate gene (Supplementary Figure S7G).

### Sequence Divergence

**FIGURE 4:**
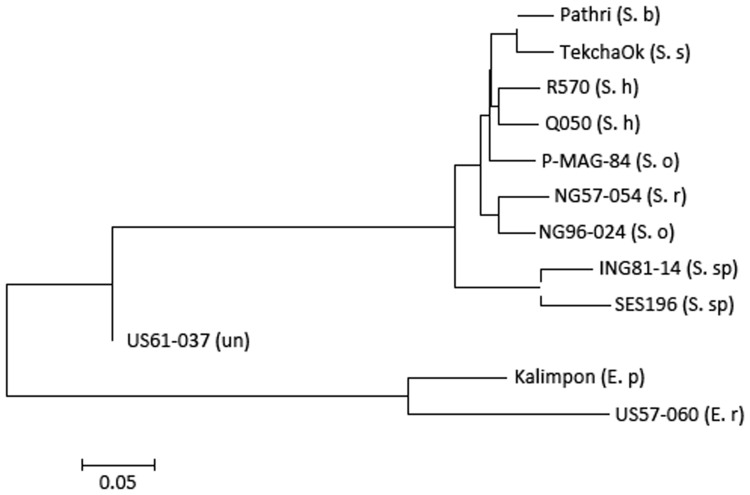
**Phylogenetic tree of the 12 accessions generated using biallelic- single nucleotide polymorphism of the 12 accessions**.

**FIGURE 5:**
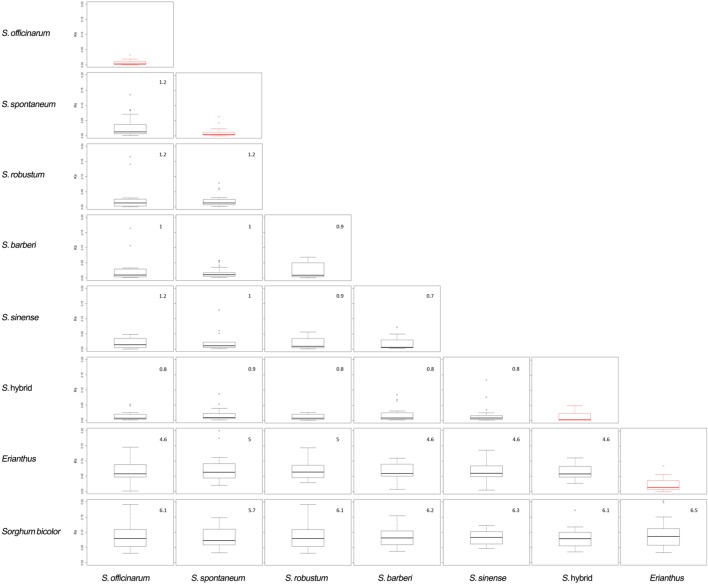
**Divergence plots between different accessions**. The red boxplot shows the Ks between two accessions from same species.

Phylogenetic analysis of the 12 accessions revealed that the two *Erianthus* accessions grouped into one cluster, and all the *Saccharum* spp. were also grouped into another cluster (**Figure 4**). The unknown accession (US61-037) was closer with *Saccharum* species than with the two *Erianthus* species, most likely a hybrid clone derived from a cross between the *Saccharum* and *Erianthus*. To estimate divergence among the 12 accessions in *Saccharum* complex, the substitution rates of synonymous (Ks) for 25 homologs (Supplementary Table S5) among the 12 accessions were calculated between each pair of accessions. As expected, the Ks value was smaller between the two accessions within the same species (e.g., two *S. officinarum* accessions, two *Erianthus accessions* and two *S. spontaneum* accessions) compared to Ks value between two different species (**Figure 5**). Based on the common synonymous substitution rate for grass lineage (Gaut et al., 1996), the divergence times between sorghum and the 12 accessions in *Saccharum* complex were relatively long, ranging from 5.7 million years ago (MYA) (with *S. spontaneum*) to 6.5 MYA (with *Erianthus* species). The divergence times between *Erianthus* and all *Saccharum* species were estimated as 4.6–5 MYA. Within the *Saccharum* species, the divergence time between each different species was estimated to be 0.7 (between *S. Sinense* and *S. Barberi*) to 1.2 MYA (**Figure 5**).

## Discussion

### Target Enrichment Sequencing

In this study we used an in-solution hybridization based enrichment strategy to reduce the genome complexity of *Saccharum* complex and enriched widely distributed target genomic regions of 12 diverse accessions representing a big scope of species in the *Saccharum* complex for sequencing. Sequencing by methylation filtration (MF) approach can be used to enrich the genic region sequences and it has been successfully utilized to assemble gene-enriched regions of a sugarcane cultivar (Grativol et al., 2014). However, researchers have minimum control on specific target regions by using this approach especially in studies to genotype a diverse panel of germplasm in parallel. Missing data or various sequence depth at a given locus across the panel could be a problem, which is also a concern for genotyping by sequencing (GBS) approach. The probe-based enrichment approach can pinpoint specific regions to reduce the opportunity of missing data instead of conducting random sampling euchromatic regions. This probe-based enrichment approach can facilitate genotyping and cataloging the allelic variations across a large diversity panel. Multiple types of allele variants and haplotypes were captured within target region across 12 accessions. Technically, we successfully multiplexed 12 accessions for one hybridization capture reaction, which allows increasing the throughput and reducing the cost of the capture procedure. Previously, up to eight genotypes of pine trees were multiplexed for capture and sequencing (Neves et al., 2013). In this study, the multiplexing level in polyploid sugarcane was further increased to 12 and proven to be a success.

For hybridization based enrichment strategy, the probe design is a fundamental step. To fully capture the allelic variants in the *Saccharum* complex, 55,946 120-mer probes were designed from sorghum genome and sugarcane unigene set, targeting collectively a total of 6 Mb region. The capture efficiency of probes designed from sugarcane sequences was slightly higher than those from sorghum sequences (Supplementary Figure S2B). We observed more than 80% of the probes with MSD >30 in *Saccharum* spp. and only 67% probes with MSD > 30 in the two *Erianthus* species, which provided enough data per locus for variant calling and genotype inference. The capture efficiency bias between *Saccharum* species and *Erianthus* species was mostly due to their different sequence similarity to sorghum reference genome used for alignment. The *Saccharum* species was more closely related to sorghum than *Erianthus* species. The capture efficiency was significantly higher for probes designed from CDS regions than those from non-CDS regions (Supplementary Figure S3G), suggesting CDS regions are more conserved across different species or genotypes and are preferable in probe design to target corresponding regions across a diverse collection of samples. The probes with read depth of more than 10,000 could be targeting repetitive regions and generating non-uniquely mapped reads.

The sequence features of all the probes and their capture efficiency were further evaluated to determine credentials of good probes in target enrichment sequencing approach. In our study, the probes with a 30–60% GC content had optimal coverage which was similar to the previous report on wheat (Saintenac et al., 2011) but wider than the range of 30–40% previously reported (Bundock et al., 2012), which may be due to the different cut-off criteria for evaluation or different stringency during hybridization. The hairpin score showed a weak influence on MSD compared to the dimer score, PHFE and PMFE, respectively (Saintenac et al., 2011). In this study, a linear model was developed based on the probe sequence features to predict capture efficiency and subsequent variant calling, which could be recommended for use in probe design in experiments of target enrichment sequencing of other related species.

### Genomic Variant Calling Pipelines in Polyploid Species

Bioinformatics analysis on NGS data is critical for reliable genomic variants calling. We established a tailored pipeline for SNP calling for the polyploid *Saccharum* complex after comparison of different programs and options. A very well assembled reference genome is essential for NGS short reads alignment and other downstream variants calling and annotation. Since a direct reference genome is not yet available for *Saccharum* species, the choice of reference would be challenging. In our study, four references including the sorghum genome v3.1, the assembly of the sequence reads, probe sequences, and a unigene set assembled from in-house RNA-seq data were compared to identify the optimal reference for alignment. Based on the alignment rate, we found that sorghum genome was a preferred reference specifically considering its assembly into chromosomes and its thorough annotations (Paterson et al., 2009). The sugarcane transcript assembly had relatively low alignment rate due to some reads containing extended intron region (Supplementary Figure S1) thus causing significant mismatch and non-alignment. When the *de novo* assembly (>200 bp) from all reads was used as reference, the alignment rate was relatively low as well (**Table 1**), due to the removal of singletons or contigs less than 200 bp in length. When assembly with a lower length cut-off (60 bp) was used, the alignment reads increased to 98%. However, to facilitate the downstream variants distribution and annotation analysis, the sorghum genome was selected as the reference since the alignment rate (83%) was highly acceptable. The high level of nucleotide similarity (97%) between *Saccharum* and sorghum coding region demonstrated the premise that the sorghum genome can serve as a good reference.

Variant calling programs built upon different models and various aligners would differentially identify potential polymorphisms because many programs are optimized on a diploid model like human. The differences were demonstrated in several polyploids, such as *Brassica napus* and peanut (Clevenger et al., 2015). Thus it is necessary to choose the programs that work effectively for the sequencing data of *Saccharum* complex. For read alignment, we evaluated three popular aligners; BWA-mem, Bowtie2 and MOSAIK. BWA-mem and Bowtie2 outperformed MOSAIK in terms of percentage of mapped reads. Additionally, BWA-mem has been shown to have slightly higher number of reads alignment than Bowtie 2. However, BWA identified more uniquely mapped reads to sorghum genome than Bowtie 2 and MOSAIK indicating that BWA was more sensitive for the uniquely mapped reads calling. By evaluating the three aligner settings, we noticed that BWA-mem uses different seed extension algorithm, which allowed 4% of mismatch, while other aligners only allow mismatch at low sequence quality sites, which were trimmed in this study. Considering (1) the sequence identity of 95% between the sorghum genome and sugarcane sequences in the genic region (Wang et al., 2010) and (2) only uniquely mapped reads to be used for SNP calling, which minimized the misplaced reads for SNP calling, we chose BWA-mem for reads alignment for further analysis. With the nearly 90% SNP validation rate (SNPs not validated were mostly single dose SNPs), we would recommend that BWA-mem aligner is suitable for alignment of sugarcane sequences at genic regions to sorghum genome for SNPs calling.

The choice of sequence variant caller might lead to variability in the variants called. SAMtools and GATK are widely used callers, and often used in combination for variant calling (Brownstein et al., 2014). The comparison of these two variant callers demonstrated low concordance by using parallel analysis of same dataset from four human family exome sequencing (ORawe et al., 2013). For SNP calling, we compared three programs, SAMtools, Freebayes, and GATK. All three callers used Bayesian method that incorporated several factors including preprocessing alignment, modeling multi-allelic loci and genotype likelihood model. SAMtools was developed to call variants for haploid or diploid with a low likelihood to call heterozygote due to a prior setting (Li, 2014). The GATK and Freebayes could handle different ploidy level, which could be used for the variant calling in polyploid species (McKenna et al., 2010; Garrison and Marth, 2012). However, Freebayes performed very conservatively in calling SNPs in this study (lowest number of variants called), which is inconsistent with the results reported previously on peanut (Clevenger et al., 2015). Typically, GATK offered the best practice guidelines including base quality score recalibration and variant-calling score recalibration for SNPs calling; and local realignment for the InDels calling. However, as sugarcane was a non-model species, there was no training dataset publicly available for recalibration. To control the accuracy, stringent hard-filtering criteria were applied to the variants call-sets generated from GATK in this study. SNPs called by the three callers were randomly selected for validation using Sanger re-sequencing. The validation rates were approximately 90% from all three callers. Even the validation rate of the SNPs concordantly called by all three callers was not higher than that called by the individual callers. Therefore, to maximize the sequence variants calling, specifically for SNP calling, we suggest using the combination of all three callers. The follow up SNP validation on segregating population could be used to benchmark the different computational pipelines as well as to select the valid SNPs from the computationally called SNPs for future SNP genotyping of *Saccharum* complex.

### Allelic Variants in Polyploid *Saccharum* Species

Genetic mapping in sugarcane has been a big challenge owing to the huge number of chromosomes and uncertainty of chromosomal recombination, allele dosage, and their inheritance pattern in the complex polyploids. Normally, SDM were identified as valuable marker source for linkage analysis in polyploid species, such as sweet potato (Kriegner et al., 2003), eucalyptus (Yin et al., 2001), potato (Bonierbale et al., 1988), and apple (Hemmat et al., 1994). Over the past decades, several linkage maps of sugarcane were constructed (Hoarau et al., 2001; Ming et al., 2002; Aitken et al., 2005; Garcia et al., 2006; Raboin et al., 2006; Somers et al., 2007; Andru et al., 2011). However, all of them were far from saturation, due to huge number of chromosome (over hundreds) and not enough SDMs. It was noticed that the number of SDM decreased with increasing ploidy level (Garcia et al., 2006). In our dataset, the highest number of SDM was identified in an unknown accession (US61-037), which is a hexaploid. The other hexaploids such as the two *Erianthus* species and a *S. spontanum* species however have had similar percentage of SDM as other accessions with higher ploidy levels (Supplementary Table S1; **Figure 3**). We further identified 524 common SDMs between our dataset and another SNP dataset called for a mapping population with 173 individuals (unpublished data). Sixty of the 524 SNPs were confirmed as SDMs in the bi-parental F1 population. The rest of the 524 SDMs were mostly from *Erianthus* species or other different genetic background. The large number of SNPs identified in this study included SDM, which is a magnificent genetic resource to the sugarcane community. In addition, we have identified several other structural variances such as InDels, CNVs, and PAVs providing the first systematic study across the *Saccharum* complex. Identification of InDels proved to be more challenging than SNPs (Albers et al., 2011). There was a huge variability in InDel identification by the three callers. To be conservative, concordant InDels identified by all three callers were subjected to further analysis in this study. We found that the total number of InDels with a length of not a multiple of 3 were less abundant in the coding regions as compared to genome-wide suggesting that the coding regions are under intensive purifying selection. Similar results were reported in sorghum genome as well (Zheng et al., 2011).

We have identified a large number of gene CNVs and PAVs among *Saccharum* species and between two *Erianthus* species using stringent criteria. The genes involved in CNV/PAV usually belong to a gene family, where each member is functionally redundant and not associated with lethality or loss of fitness (Swanson-Wagner et al., 2010). The gene CNV may result from genome haplotype-specific tandem duplication, non-allelic paralogous duplication, and recombination (Innan and Kondrashov, 2010; Swanson-Wagner et al., 2010). Tandem duplications were often observed in sugarcane genome (Wang et al., 2010) and other species such as maize, sorghum, *Setaria italica* (Springer et al., 2009; de Setta et al., 2014). Recombination-based mechanisms have been reported in maize genome (Swanson-Wagner et al., 2010). However, gene PAVs might arise from fractionation process due to polyploidization (Woodhouse et al., 2010). In this study, we used comparative sequence read alignment depth to estimate the gene CNV and PAV. Many factors complicate PAV and CNV calling such as ploidy level, read number of each sample, alignment rate reflecting sequence homology level between samples, and sorghum whose sequences were used for probe design. To be conservative, very stringent criteria were applied to call the PAV and CNV, so the numbers of PAV/CNVs may still be underestimated. Some species-specific gene CNV/PAVs were identified, which may be associated with speciation and species-specific traits. The CNV/PAVs identified in this study after further validation will contribute to future gene evolutionary and gene/trait association studies in *Saccharum* complex.

### Genotype and Haplotype Calling

Sugarcane is a vegetatively propagated outcrossing species and thus is presumably very heterozygous. For a given SNP locus, it is likely to be specifically heterozygous given its high polypoidy level. In this study, the genotype at each locus or the allele dosage was called based on the ploidy level estimated for each accession and the sequence alignment depth. The results indicated that approximately 18% (NG96-024) to 29% (TekchaOk) SNP loci among *Saccharum* spp. were heterozygous while the rest were homozygous. The low portion of heterozygous loci could be due to the minimum locus size, single nucleotide instead of a long stretch of gene allele to harbor many nucleotide polymorphisms and/or due to the low outcrossing rate of the species. Sugarcane exhibited 10 cM-long linkage disequilibrium (Jannoo et al., 1999). The outcrossing rate of some sugarcane cultivars can be as low as 1.5% (Melloni et al., 2014).

Haplotype phasing has been a powerful approach for gene mapping. Many methods have been developed for haplotype phasing and assembly (Lippert et al., 2002; Schwartz, 2010), although most of them have been done in diploid individuals. In this study, haplotype was identified for the first time in polyploid sugarcane genome. The large number of bi-allelic SNP variants were parsed into haplotype blocks with the longest size of 2,453 bases. The length of the haplotype could be affected by the density of SNPs. We specifically characterized the haplotype patterns of 406 candidate genes, which provided great information in understanding the allelic variation of polyploid species. The accuracy of haplotype could be further validated by sequencing clones of large insert genomic DNA libraries.

### Divergence of the *Saccharum* Complex

The divergence and speciation within the *Saccharum* complex has been an interesting topic for decades. It was reported that the divergence between sorghum and *Saccharum* spp was 5, 7.7, or 8–9 MYA in different studies based on different small sets of sequences from selected gene or region (Al-Janabi et al., 1994; Jannoo et al., 2007; Wang et al., 2010). The Ks values for 19 pairs of sucrose synthase genes among three *Saccharum* species (Zhang et al., 2013) are similar to our results. In this study, the divergence time of the *Saccharum* complex was estimated based on genome-wide targeted genes. The *Saccharum* species and sorghum was predicted to have diverged 5.7-6.1 MYA, quite similar to previous reports (Al-Janabi et al., 1994; Jannoo et al., 2007; Wang et al., 2010). The divergence between the *Saccharum* and *Erianthus* was first estimated as approximately 5 MYA in our study. These relative divergence times further support the phylogenetic relationship among the 12 accessions.

## Conclusion

In this study, a hybridization-based target enrichment sequencing approach was used to investigate the allelic variation of 12 accessions representing variety of different species in *Saccharum* complex. A pipeline for variants calling within polyploid genome was established and demonstrated to be effective. A large number of genetic variations including SNPs, InDels, gene CNVs and gene PAVs were identified in *Saccharum* species and *Erianthus* species indicating the existence of a high level of genetic variations within the *Saccharum* complex. The hybridization-based target enrichment sequencing approach proved to be an efficient way to discover natural allelic variation in highly polyploid and heterozygous species, which would be costly with whole genome shotgun sequencing. The results can be applied to identify different gene alleles and allele variants recombination that is responsible for adaptation to different environmental conditions and other important traits of interests.

## Author Contributions

JW conceived and designed the experiments. JS, MR, LN, and JT performed the experiments. JS, XY performed data analysis. JCC, JZ provided materials and reference. JS, XY, and JW drafted the manuscript. JW, MR, LN, JT, JZ revised the manuscript. All authors read and approved the final manuscript.

## Conflict of Interest Statement

The authors declare that the research was conducted in the absence of any commercial or financial relationships that could be construed as a potential conflict of interest.

## Footnotes

1 ftp://occams.dfci.harvard.edu/pub/bio/tgi/data/Saccharum_officinarum

2 http://www.agilent.com/

3 http://sourceforge.net/projects/bio-bwa/files/

4 http://sourceforge.net/projects/bowtie-bio/files/bowtie2/2.2.5/

5 https://code.google.com/p/mosaik-aligner/

6 http://trinityrnaseq.github.io/

7 http://www.brown.edu/Research/Istrail_Lab/

8 https://github.com/jdpr100/Sugarcane-target-enrichment-analysis.git

9 http://biotools.umassmed.edu/bioapps/primer3_www.cgi

10 www.DnaBaser.com

11 http://phytozome.jgi.doe.gov/pz/portal.html

12 http://bioinfo.cau.edu.cn/agriGO/

13 www.phytozome.org

14 ftp://ftp.ebi.ac.uk/pub/software/clustalw2/

15 http://tree.bio.ed.ac.uk/software/figtree/
