# Supplementary material for: Natural Allelic Variations in Highly Polyploidy Saccharum Complex
Source: Front Plant Sci. 2016 Jun 8;7:804. doi: 10.3389/fpls.2016.00804 (PMC4896942; doi:10.3389/fpls.2016.00804)
Supplement: Supplementary file 3 [file Data_Sheet_1.DOC]

Supplementary Materials

**Natural allelic variations in highly polyploid *Saccharum* complex**

Jian Song, Xiping Yang, Marcio F. R. Resende Jr., Leandro Gomide Neves, James Todd, Jisen Zhang, Jack Comstock, Jianping Wang^*^

^*^Corresponding author, Email: [wangjp@ufl.edu](mailto:wangjp@ufl.edu)

**1. Supplementary data**

**The phenotypic characteristics**

The phenotypic traits collection of these 11 accessions were conducted as described by Todd et al (Todd et al., 2014). A total of 10 traits were phenotypically evaluated before and after ratoon harvesting, including stalk height (cm), stalk diameter (cm), leaf width (mm), leaf length (cm), total fresh weight (kg), total stalks (N), total Brix (%), internode (cm), dry biomass (kg) and orange rust resistance (sporolating or non-sporolating after inoculation).

The 11 accessions differ in numerous morphological and agronomic traits (Fig SS1). As noble cane, *S. officinarum* accessions (P-MAG-84, NG96-024) and hybrid cultivar (Q050) had significantly larger stalk diameter, higher stem Brix content and leaf width than *S. spontaneum* (SES196). The *S. spontaneum* line (IND81-14) had highest number of tillers of the 11 accessions and the two *S. spontaneum* accessions have greater rust resistance when compared to *S. officinarum* accessions and hybrid (Q050) indicating the typical nature of this wild progenitor of sugarcane. *S. sinense* (TekchaOk) has relatively high total Brix, broad leaves, high stalk height and thick stalks. The *S. robustum* accession (NG57-054) is similar to the *S. barberi* accession (Pathri) and both had similar height, stalk diameter, leaf width, leaf length etc. The *Erianthus* accession (US57-060) is morphologically closer to *S. spontaneum* (IND81-15) than to the other species, such as higher stalk number, smaller stalk diameter and narrower leaves. In terms of orange rust resistance, *spontaneum* accessions (SES196, IND81-15) and *Erianthus* accessions (Kalimpon, US57-060) showed resistance to orange rust disease, while the rest of the accessions were susceptible to orange rust. (Fig. SS1)

**Chromosome number estimation**

To estimate the ploidy level of each of the 12 accessions, the chromosome numbers and genome size were estimated using the flow cytometry method according to the procedure described by Dolezel with slight modification (Dolezel et al., 2007). Briefly, the young leaves that newly emerged from leaf whorl were taken from plants. Fifty mg fresh young leave tissue of each genotype was chopped by itself and also with the internal standard in the nuclei isolation buffer (Partec Gmbh-Munster, Germany) followed by filtering with 40 um nylon filter. A volume of 1.6 ml 4’, 6-diamidino-2-phenylindole (DAPI) was added to the filtration and incubated for 5 minutes for DNA staining. The suspension of nuclei was analyzed using Partec-PA flow cytometer (Partec Gmbh-Munster, Germany). Three *Saccharum* genotypes used as internal standard included a *S. officinarum* accession of Yellow Caledonia (2n=80, 7.85 pg/2C), a *S. spontaneum* accession of SES 208 (2n=64, 6.89 pg/2C) and a *S. robustum* accession of NG 77-084 (2n=80, 7.82 pg/2C) (da Silva et al., 1995; Zhang et al., 2012). Yellow Caledonia (2n=80) belonging to *S. officinarum* spp. was used as internal standard to estimate the ploidy level of the two *S. officinarum* spp., P-MAG-84 and NG96-024. Since there was no species-specific internal reference available for *S. barberi*, *S. sinense*, hybrids and *Erianthus*, Yellow Caledonia (2n=80) was chosen to estimate chromosome number for these species. The fluorescence signals were used to calculate chromosome number and genome size according the method described by Dolezel et al (Dolezel et al., 2007).

The fluorescence intensities of P-MAG-84 and NG96-024 were the same as G0/G1 peak of Yellow Caledonia and the chromosome number was estimated as 80 (Supplementary Table S1). The two *S. spontaneum* (SES196, IND81-14) chromosome numbers were estimated as 64 and 48 based on the ratio of G1 peak using SES 208 (2n=64), respectively. The G1 peak of *S. robustum* (NG57-054) was the same with internal standard NG 77-084 (2n=80) and therefore, the chromosome number of NG57-054 was estimated as 80.

**References**

Doležel, J., Greilhuber, J., and Suda, J. (2007). Estimation of nuclear DNA content in plants using flow cytometry. *Nat. protoc*. 2:2233-2244.

da Silva, J., Honeycutt, R. J., Burnquist, W., Al-Janabi, S. M., Sorrells, M. E., Tanksley, S. D., et al. (1995). *Saccharum spontaneum* L.‘SES 208’genetic linkage map combining RFLP-and PCR-based markers. *Mol. Breed*. 1:165-179.

Todd, J., Wang, J., Glaz, B., Sood, S., Ayala-Silva, T., Nayak, S. N., et al. (2014). Phenotypic characterization of the miami world collection of sugarcane and (*Saccharum* spp.) and related grasses for selecting a representiative core. *Genet. Resour. Crop Evol*. 61:1581-1596.

Zhang, J., Nagai, C., Yu, Q., Pan, Y., Ayala-Silva, T., Schnell, R. J., et al. (2012). Genome size variation in three *Saccharum* species. *Euphytica*185:511-519.


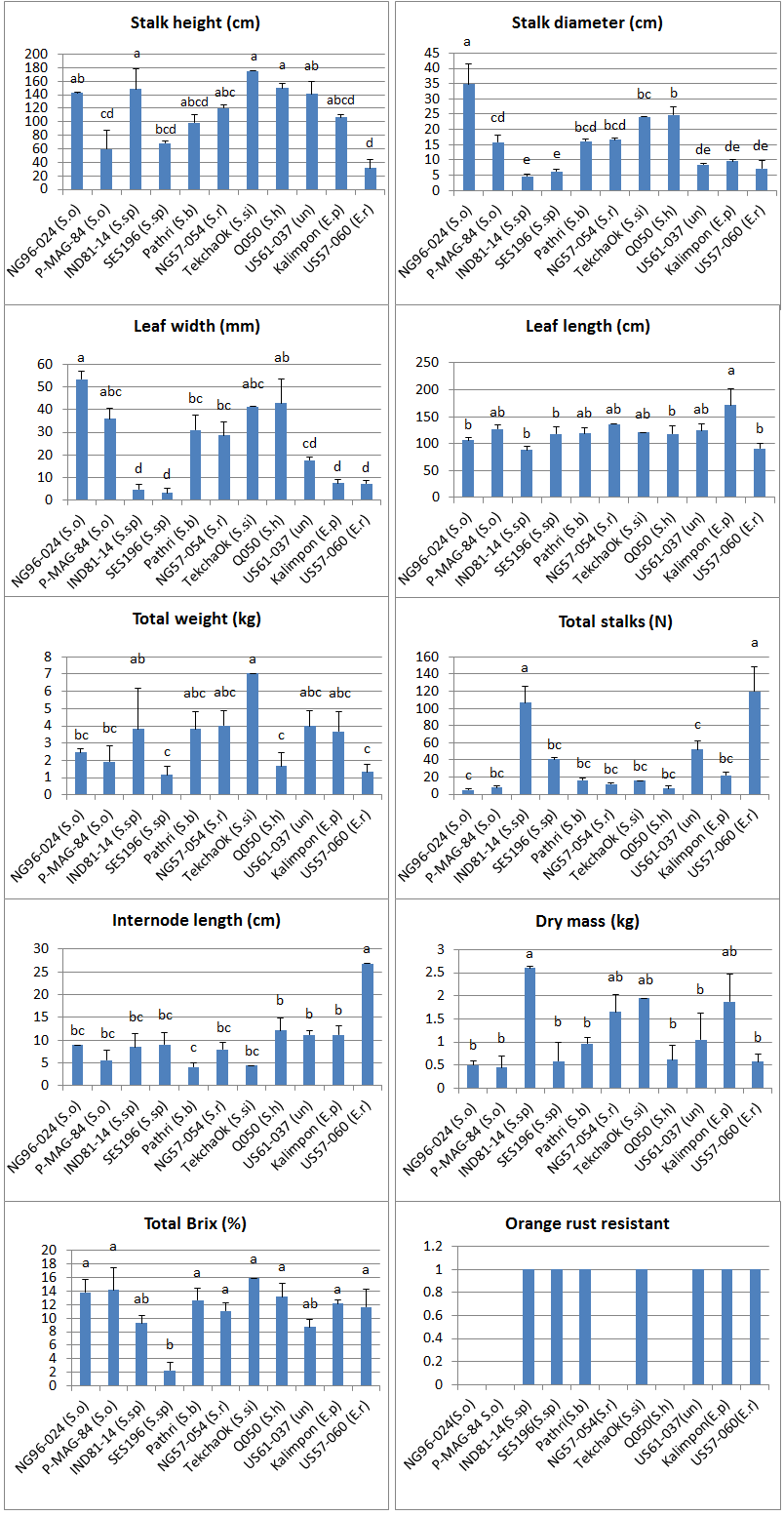


Fig. SS1 The measurement of phenotypic traits of 11 accessions. The statistical comparisons among 11 accessions were performed for the following measurement stalk height (cm), stalk diameter (cm), leaf width (mm), leaf length (cm), total fresh weight (kg), total stalks (N), total Brix (%), internode (cm) and dry biomass (kg). Different letters on top of bars indicated group which was assigned based on Least Significant Difference (LSD) test with Bonferroni adjusted P value <0.05. For the orange rust resistant, 1 represented accessions resistant to orange rust and 0 standed for accessions susceptible to orange rust.

# 2 Supplementary Figures and Tables

## 2.1 Supplementary Figures


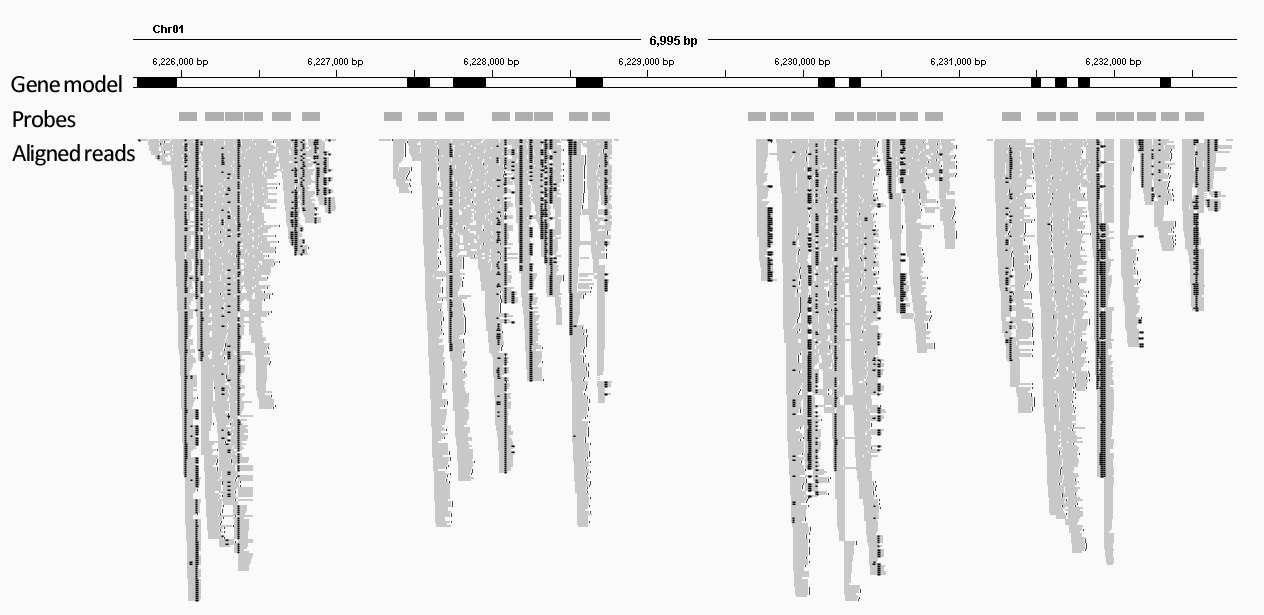


Supplementary Figure S1. Read alignment to sorghum gene model

Illustration of reads mapped to sorghum gene model visualized by IGV program demonstrating the alignment location of reads from NG96-024 accession. The top black box showed the exon region of the locus Sobic.001G080700. The middle blue box indicated the probes designed from this locus.


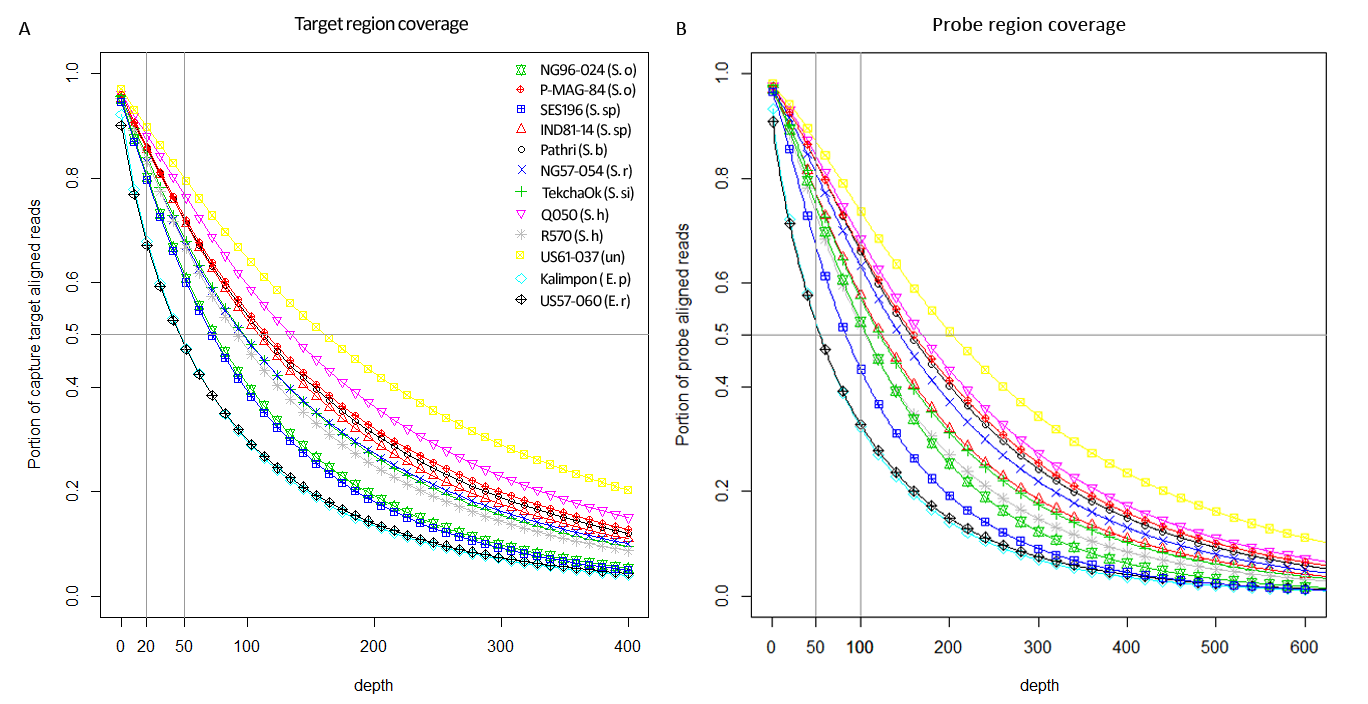


Supplementary Figure S2. The cumulative percentage of target regions and probes plotted against reads depth of coverage. (A) The cumulative coverage proportion of target regions. The target capture regions were defined by adding 100-bp up and downstream of each probe location on the sorghum reference genome. The cumulative coverage of target region describing the fraction of targeted bases that were covered reads from target enrichment sequencing was plotted. The lines with different colors represented different accessions. (B) The cumulative coverage proportion of probes. The cumulative percentage of probes describing the fraction of each base from 55,946 probes covered by reads was plotted which show the read depth of coverage for each probe.


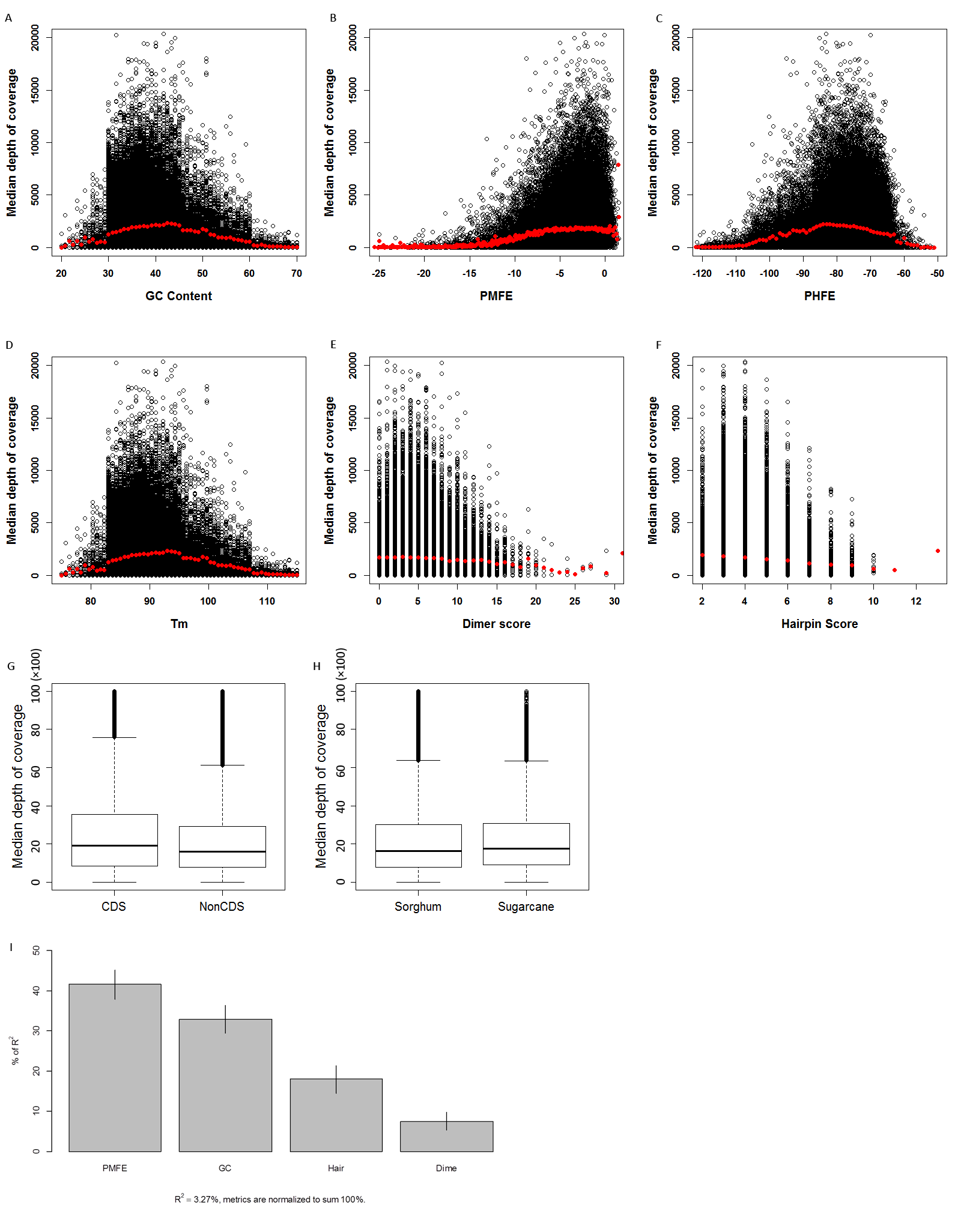


Supplementary Figure S3. Influence of probes on capture efficiency. (A-F) Influence of probe properties, Tm, melting temperatures (PMFE), probe minimum folding energy (PHFE), and probe hybridization free energy, on capture efficiency. (G) Influence of probes from sorghum gene coding regions and non-coding regions on read depth of coverage. (H) Influence of probes from sorghum genome and sugarcane unigene on read depth of coverage. (I) Relative importance estimated for the probe parameters used in the linear model for probe capture efficiency. The analysis was performed with 95 % bootstrap confidence interval using method LMG in Relaimpo package in R 3.0


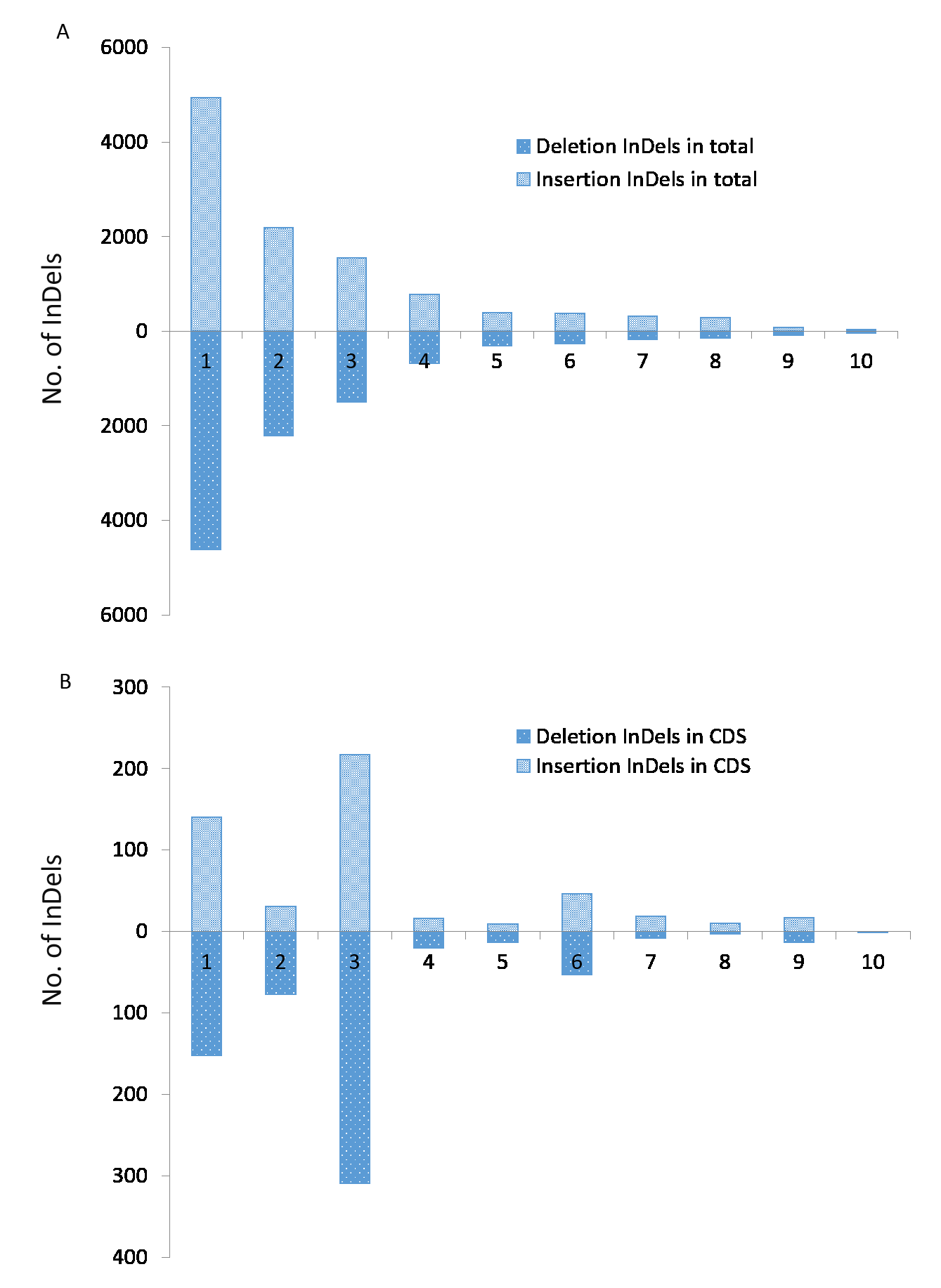


Supplementary Figure S4. The number of InDels identified at difference sizes from 1 to 10 bp according to the sorghum genome. (A) The figure showed the number of InDels with different length in the coding region according to sorghum gene model. (B) The figure showed the number of genome-wide InDels with different length


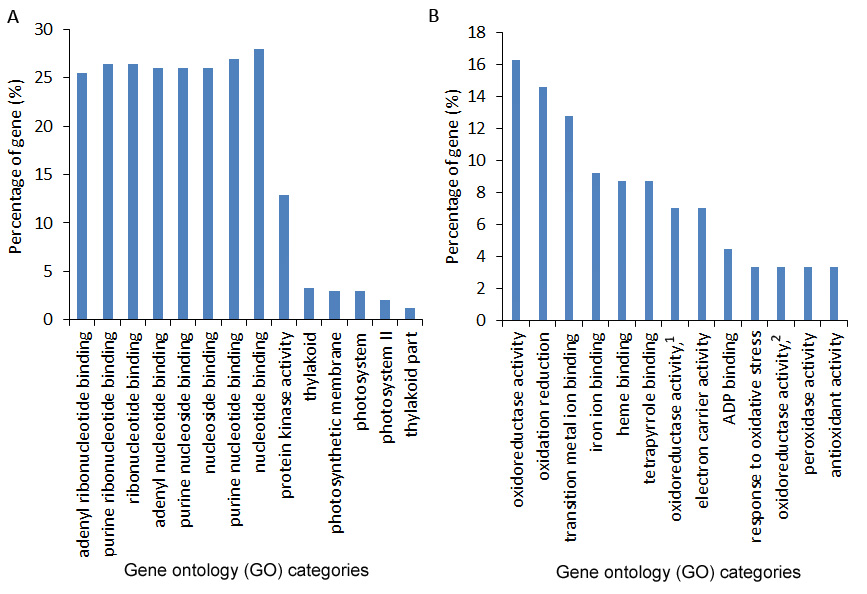


Supplementary Figure S5. Gene ontology (GO) enrichment analysis of the genes involved copy number and presence/absence variation. (A) Enriched GO terms in genes with copy number variations (CNV) within 12 accessions (B) Enriched GO terms in genes with presence/absence variations (PAV) within 12 accessions. Note: 1. oxidoreductase activity, acting on paired donors, with incorporation or reduction of molecular oxygen; 2. oxidoreductase activity, acting on peroxide as acceptor


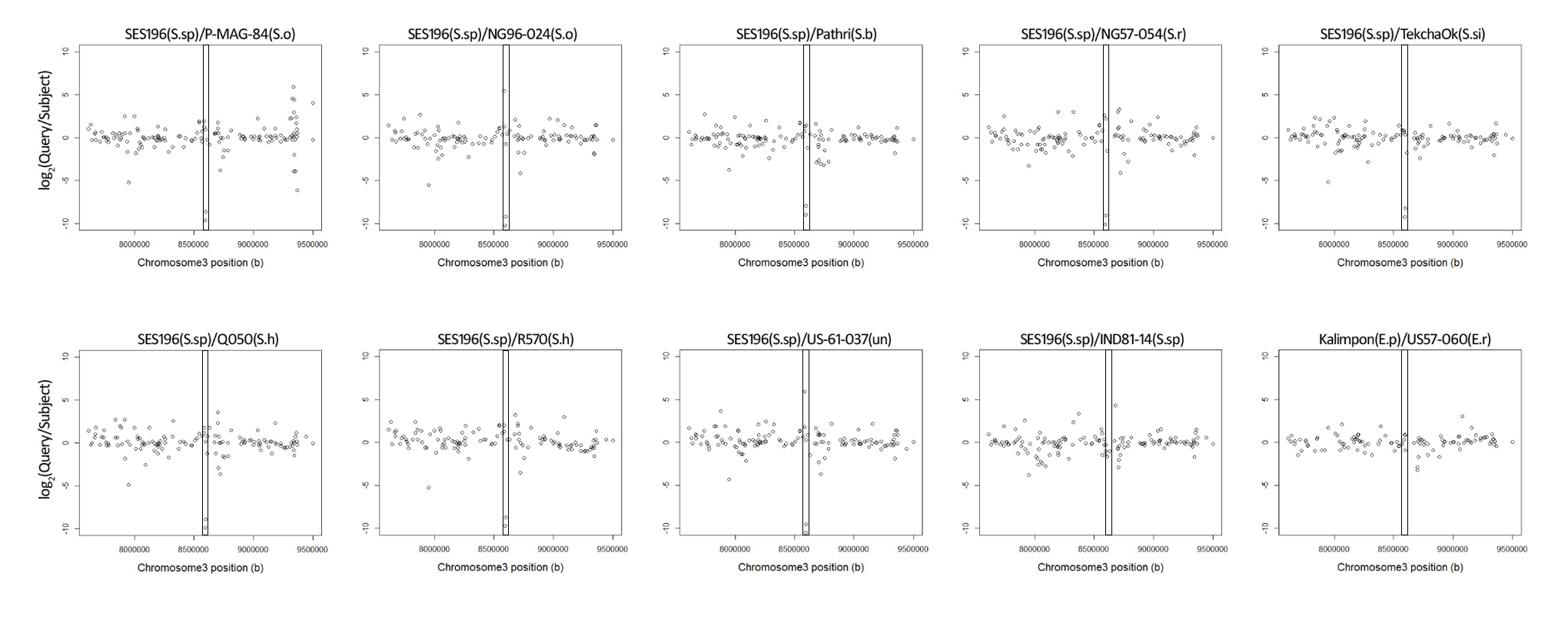


Supplementary Figure S6. Illustration of a *Saccharum spontanum* - specific copy number variation (CNV). Plot of log_2_ ratios between the number of normalized mapped reads from query accession and that of subject accession for the genes within 2Mb window size on sorghum chromosome 3 to demonstrate a *Saccharum spontanum* - specific copy number variation (CNV). The log_2_ ratio between *Saccharum spontaneum* accession (SES196) and the rest of *Saccharum* species showed two of the *Saccharum spontaneum-* specific down-CNVs. The black bar indicated the down-CNVs region on the sorghum chromosome 3.


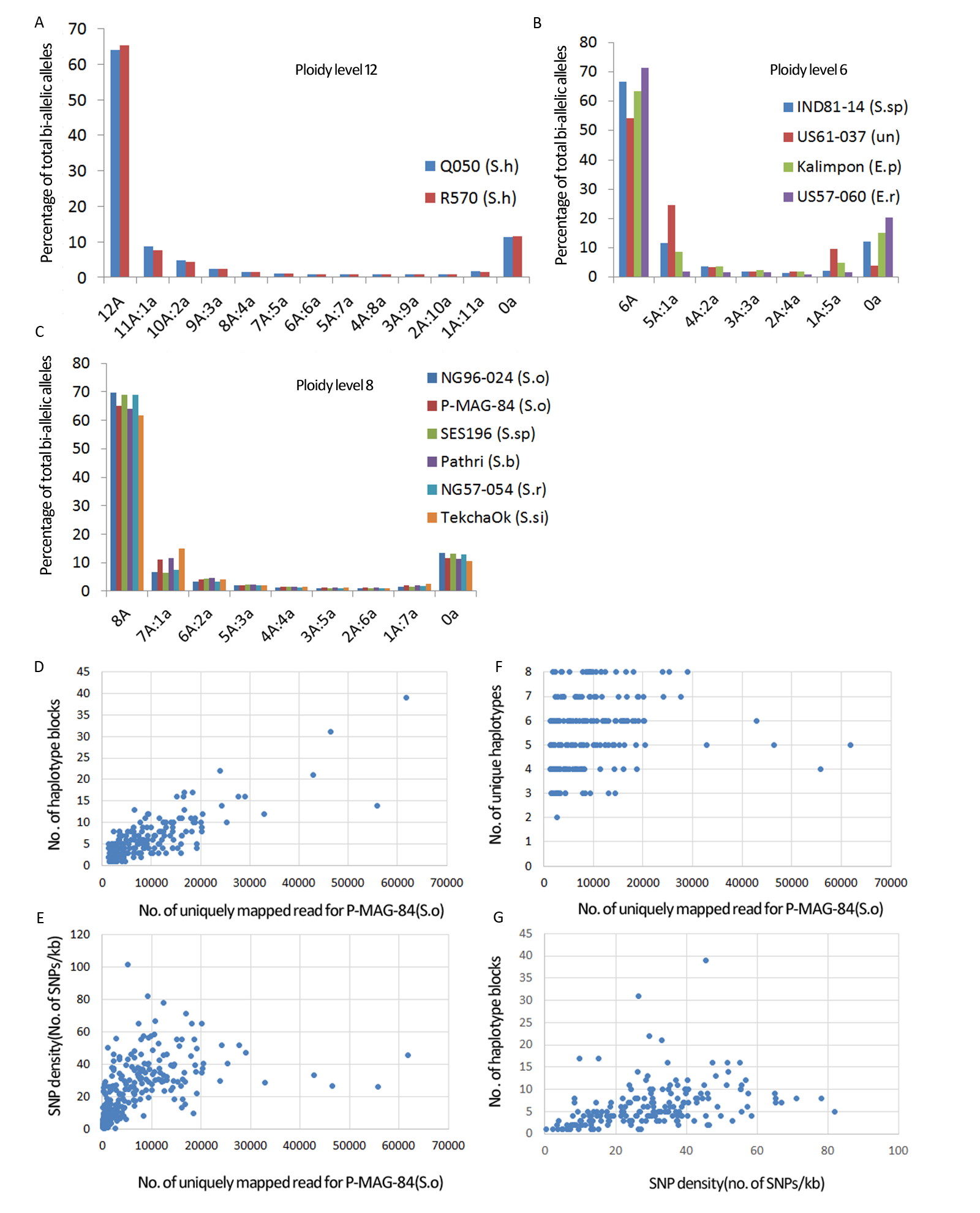


Supplementary Figure S7. Genotype and haplotype calling. (A-C) The percentage of genotypes called at different ploidy level using biallelic SNPs for each of the 12 accessions. “A” represented the reference allele and “a” represented the alternate allele. (D-G) The relationship among the number of uniquely mapped reads, number of SNPs, and number of haplotype block. The number of uniquely mapped reads from one *Saccharum officinarum* accession (P-MAG-84) plotted against the number of haplotype blocks for 406 candidate genes (D), the SNP density for each candidate gene (E) and the non-duplicated haplotype number for each candidate gene (F). The SNP density for each candidate gene plotted against the number of haplotype blocks (G).

**2.2 Supplementary Tables**

Supplementary Table S1. Genome size and ploidy level estimated in 11 accessions

| Clone name | Ploidy level | No. of estimated Chr. | DNA content (pg/2C) | Genome size (Gb/2C) |
| --- | --- | --- | --- | --- |
| NG96-024 (S. o) | 8× | 80 | 7.85±0.08 | 7.68 |
| P-MAG-84 (S. o) | 8× | 80 | 7.85±0.06 | 7.68 |
| IND81-14 (S. sp) | 6× | 48 | 5.41±0.07 | 5.3 |
| SES196 (S. sp) | 8× | 64 | 6.89±0.07 | 6.74 |
| Pathri (S. b) | 8× | 80 | 7.89±0.1 | 7.82 |
| NG57-054 (S. r) | 8× | 80 | 7.95±0.05 | 7.79 |
| TekchaOk (S. si) | 8× | 80 | 8.26±0.21 | 8.09 |
| Q050 (S. h) | 12× | 110-120 | 9.67±0.2 | 9.5 |
| R570 (S. h)* | 12× | 115 |  | 10 |
| US61-037 (un) | 6× | 60 | 6.76±0.16 | 6.65 |
| Kalimpon (E. p) | 6× | 60 | 6.68±0.13 | 6.55 |
| US57-060 (E. r) | 6× | 60 | 6.59±0.13 | 6.46 |

* refer to D'Hont (2005). S. o: *Saccharum* *officinarum*; S. sp: *Saccharum* *spontaneum*; S. b: *Saccharum* *barberi*; S. r: *Saccharum* *robustum*; S. si: *Saccharum* *sinense*; S. h: *Saccharum* hybrid; un: unknown; E. p: *Erianthus procerum*; E. r: *Erianthus* *rufipilu*

Supplementary Table S2. Probes designed for target enrichment experiment (Submitted separately due to big size)

Supplementary Table S3. Primers list used for SNPs validation

| Primer name | Forward primer | Reverse primer |
| --- | --- | --- |
| SV_c01-1 | ACAAGGGTGGAACGTGAAAG | GCCAAGGCTGAACTTGAGAA |
| SV_c01-2 | GCGAAGCTTACAAACGATCC | TGACTGCAAGAATGTTCATGGT |
| SV_c03-1 | TGTGGTCAAAGGGGGATTTA | TTGATGCAGGAATGCACAGT |
| SV_c07-1 | GCTTTGTCCGGAGAGTTCAG | GGAGCGTAACCTCCTGTCTG |
| SV_c04-1 | AATTCCGTGAGAGGTGCAGT | ACATTTGATCGGCATCCTTC |
| SV_c05-1 | CCCAATGTGGATTTGATGCT | CTTCCTGATGGCATTGATGA |
| SV_c09-1 | TTCGCATGGAGAACCTTACA | ATTTGGCTGGGCGTATGTTA |
| SV_c09-2 | AAGCCTACACAGGCTGATTCAT | TTTCAGTTTAGCGCTCTTTGC |
| SV_c10-1 | CGACAAGCTGCACATTGATT | TCCTTGCCACATGCAATACA |
| SV_c02-1 | GTGACCGTTCACGAGTGTTG | AGGTTACCAGGGATGTCTGC |
| SV_c06-1 | TTTACAATGTGGTGGCATCC | ATGTCTGGAGTTCGGTGAGC |
| SV_c08-1 | GCAATTGCAAAGCTTCTGGT | TATGTTACCTCTCGTCAGCT |
| SV_c08-2 | CATGGGAAGCCAAAGTGAAT | ATCGGCATTGGGGAATTAGT |

Supplementary Table S4. Validation of SNPs called from three different callers

|  | Samtools | Freebayes | GATK |
| --- | --- | --- | --- |
| Total No. of SNPs for validation | 69 | 31 | 57 |
| No. of specific SNPs | 47 | 9 | 35 |
| No. of common SNPs | 22 | 22 | 22 |
| Total No. of false positive SNPs | 9 | 3 | 6 |
| No. of False positive SNPs in specific category | 6 | 0 | 3 |
| No. of False positive SNPs in common category | 3 | 3 | 3 |
| Percentage of positive SNP overall (%) | 87.0 | 90.3 | 89.5 |
| Percentage of positive SNPs in specific category (%) | 87.2 | 100 | 91.4 |
| Percentage of positive SNPs in common category (%) | 86.4 | 86.4 | 86.4 |

Supplementary Table S5. SNP density within gene regions according to sorghum gene model (Submitted separately due to big size)

Supplementary Table S6. List of haplotype blocks and heterozygosity rates within the target region for twelve accessions

| Species name | No. of haplotype blocks | Maximum length of block (bp) | Heterozygosity rate (%) |
| --- | --- | --- | --- |
| NG96-024(S.o) | 31,360 | 1,370 | 0.43 |
| P-MAG-84 (S.o) | 38,640 | 1,526 | 0.51 |
| IND81-14 (S.sp) | 37,254 | 1,703 | 0.49 |
| SES196 (S.sp) | 32,954 | 1,264 | 0.52 |
| Pathri (S.b) | 36,242 | 1,326 | 0.59 |
| NG57-054 (S.r) | 32,972 | 1,293 | 0.45 |
| TekchaOk (S.si) | 41,708 | 2,020 | 0.70 |
| Q050 (S.h) | 39,286 | 2,124 | 0.51 |
| R570 (S.h) | 39,538 | 2,453 | 0.66 |
| US61-037 (un) | 33,230 | 2,211 | 0.96 |
| Kalimpon (E.p) | 35,871 | 1,542 | 0.66 |
| US57-060 (E.r) | 13,708 | 1,358 | 0.28 |
| S. o: *Saccharum* *officinarum*; S. sp: *Saccharum* *spontaneum*; S. b: *Saccharum* *barberi*; S. r: *Saccharum robustum*; S. si: *Saccharum* *sinense*; S. h: *Saccharum* hybrid; un: unknown; E. p: *Erianthus* *procerum*; E. r: *Erianthus* *rufipilus* | | | |
